# Environmental adaptability and stress tolerance of *Laribacter hongkongensis*: a genome-wide analysis

**DOI:** 10.1186/2045-3701-1-22

**Published:** 2011-06-14

**Authors:** Susanna KP Lau, Rachel YY Fan, Tom CC Ho, Gilman KM Wong, Alan KL Tsang, Jade LL Teng, Wenyang Chen, Rory M Watt, Shirly OT Curreem, Herman Tse, Kwok-Yung Yuen, Patrick CY Woo

**Affiliations:** 1 State Key Laboratory of Emerging Infectious Diseases, Hong Kong; 2 Research Centre of Infection and Immunology, The University of Hong Kong, Hong Kong; 3 Carol Yu Centre of Infection, The University of Hong Kong, Hong Kong; 4 Department of Microbiology, The University of Hong Kong, Hong Kong; 5 Faculty of Dentistry, The University of Hong Kong, Hong Kong

## Abstract

**Background:**

*Laribacter hongkongensis *is associated with community-acquired gastroenteritis and traveler's diarrhea and it can reside in human, fish, frogs and water. In this study, we performed an in-depth annotation of the genes in its genome related to adaptation to the various environmental niches.

**Results:**

*L. hongkongensis *possessed genes for DNA repair and recombination, basal transcription, alternative σ-factors and 109 putative transcription factors, allowing DNA repair and global changes in gene expression in response to different environmental stresses. For acid stress, it possessed a urease gene cassette and two *arc *gene clusters. For alkaline stress, it possessed six CDSs for transporters of the monovalent cation/proton antiporter-2 and NhaC Na^+^:H^+ ^antiporter families. For heavy metals acquisition and tolerance, it possessed CDSs for iron and nickel transport and efflux pumps for other metals. For temperature stress, it possessed genes related to chaperones and chaperonins, heat shock proteins and cold shock proteins. For osmotic stress, 25 CDSs were observed, mostly related to regulators for potassium ion, proline and glutamate transport. For oxidative and UV light stress, genes for oxidant-resistant dehydratase, superoxide scavenging, hydrogen peroxide scavenging, exclusion and export of redox-cycling antibiotics, redox balancing, DNA repair, reduction of disulfide bonds, limitation of iron availability and reduction of iron-sulfur clusters are present. For starvation, it possessed phosphorus and, despite being asaccharolytic, carbon starvation-related CDSs.

**Conclusions:**

The *L. hongkongensis *genome possessed a high variety of genes for adaptation to acid, alkaline, temperature, osmotic, oxidative, UV light and starvation stresses and acquisition of and tolerance to heavy metals.

## Background

In 2001, *Laribacter hongkongensis*, a novel genus and species that belongs to the *Neisseriaceae *family of β-subclass of the Proteobacteria, was discovered from the blood and empyema pus of a patient with underlying alcoholic cirrhosis [1]. Subsequently, it was observed that *L. hongkongensis *was associated with community-acquired gastroenteritis and traveler's diarrhea in human [2-5]. *L. hongkongensis* is capable of living under a variety of ecological niches. In addition to humans, *L. hongkongensis* resides in the intestines of a variety of freshwater fish, most commonly those of the carp family, including grass carps (Ctenoharyngodon idellus), bighead carps (Aristichthys nobilis) and mud carps (Cirrhina molitorella), as well as those of frogs [4,6-9]. Moreover, it can also survive and replicate as a free living bacterium in water obtained from drinking water reservoirs [10]. To survive in these ecological niches, *L. hongkongensis* needs the capability of protecting DNA damages by endogenous and exogenous metabolites and regulating the expression of a variety of genes, which makes it able to adapt to different temperatures, pH and osmotic pressures, as well as oxidative and ultraviolet light stresses.

In this article, we present an overview of the genes of the *L. hongkongensis* genome related to DNA repair and recombination and regulation of gene expression. In addition, the putative genes and mechanisms that enable *L. hongkongensis* to adapt to different temperatures, pH and osmotic pressures, as well as oxidative and ultraviolet light stresses were also presented. These genes of *L. hongkongensis* were compared to those of *Neisseria gonorrhoeae*, *Neisseria meningitidis* and *Chromobacterium violaceum*, the other three bacteria of the *Neisseriaceae* family of β-proteobacteria with complete genome sequences available [11-13]. Human is the only known reservoir and host for *N. gonorrhoeae* and *N. meningitidis*. *N. gonorrhoeae* is most commonly associated with sexually transmitted infections and *N. meningitidis* is most commonly associated with pyogenic meningitis and bacteremia. *C. violaceum* is highly versatile and can be found abundantly in multiple ecosystems, including water and soil, in tropical and subtropical regions. It is associated with infrequent but potentially fatal infections in humans.

## Results and discussion

### DNA repair

Several pathways are involved in the repair of mutagenic and cytotoxic effects of DNA damage that can arise through endogenous and exogenous stress in bacteria.

#### Damage reversion (Direct repair)

Reversion of the damaged base is the simplest DNA repair mechanism, which involves a single-step reaction by specific enzymes. Photoreactivation and alkylation repair are two of the most well-known damage reversion mechanisms. Photoreactivation is carried out by photolyase, which acts upon lesions induced by UV irradiation in a light-dependent reaction. A gene homologous to *phrB*, which encodes a photolyase, was found in the *L. hongkongensis *genome (Table 1). Alkylation repair is mediated by the enzymes alkyltransferases, encoded by *ogt *and *ada*, as well as iron-dependent dioxygenases, encoded by *alkA*, which remove added alkyl groups from duplex DNA. Genes encoding for all three enzymes could be found in the *L. hongkongensis *genome (Table 1). Since *L. hongkongensis *can survive in natural water environments and is therefore often exposed to sunlight, these enzymes may be important for protection against such DNA damage. This is in contrast to *N. meningitidis *which lacks *alk *and some meningococcal and gonococcal strains which lack photolyase activity, which may reflect the lack of light exposure in the neisserial habitat [14].

**Table 1:** Single-strand breaks repair proteins in *L. hongkongensis *and their closest homologues

Repair pathways/Types of enzymes	Gene	Protein	Function of protein encoded	CDS	Closest match organism	Amino acid Identity (%)	Best E-value
Direct repair							
	*phrB*	PhrB protein	Repairs UV radiation-induced DNA damage by catalyzing light-dependent monomerization of cyclobutyl pyrimidine dimers between adjacent bases	LHK_02646	*L. nitroferrum*	58.73	3.00E-131
	*ogt*	Ogt	Repairs alkylated guanine by transferring alkyl group at O-6 position to a cysteine residue in the enzyme	LHK_00364	*Dechloromonas aromatica*	46.67	4.00E-30
	*ada*	Regulatory protein Ada	Repairs alkylated guanine in DNA by transferring alkyl group at the O-6 position to a cysteine residue in the enzyme	LHK_00147	*Colwellia psychrerythraea*	44.29	1.00E-60
Base excision repair							
DNA glycosylases	*alkA*	AlkA	Excises damaged DNA polymer formed due to alkylation lesions by hydrolyzing deoxyribose N-glycosidic bond	LHK_01743	*Thiobacillus denitrificans*	61.95	2.00E-62
	*mutY*	MutY	Adenine glycosylase active on G-A mispairs. Also corrects error-prone DNA synthesis due to oxidized guanine	LHK_02781	*L. nitroferrum*	63.29	1.00E-92
	*ung*	UNG	Excises uracil residues arised from misincorporation of dUMP residues by DNA polymerase or cytosine deamination	LHK_00013	*L. nitroferrum*	56.14	3.00E-58
				LHK_00723	*Ralstonia pickettii*	59.26	2.00E-33
Bifunctional glycosylases	*mutM *(*fpg*)	Formamido-pyrimidine-DNA glycosylase	Recognizes and removes damaged bases. Cleaves DNA backbone to generate single-strand break at site of base removal	LHK_00316	*Neisseria flavescens*	57.25	9.00E-90
	*nth*	Endonuclease III	Apurinic and/or apyrimidinic endonuclease activity and DNA N-glycosylase activity	LHK_01218	*Methylococcus capsulatus*	72.04	1.00E-81
AP endonucleases	*xthA*	Exodeoxyribo-nuclease III	Removes damaged DNA at cytosines and guanines	LHK_02447	*C. violaceum*	67.06	5.00E-94
	*exoA *(*xthA2*)	Exodeoxyribo-nuclease	Posseses 3' to 5' exonuclease, 3' phosphatase activities and makes DNA single-strand breaks at apurinic sites	LHK_03213	*L. nitroferrum*	73.73	1.00E-108
Nucleotide excision repair							
Global genome repair factors	*uvrA*	Protein UvrA	DNA-binding ATPase, forms recognition complex composed of 2 UvrA and 2 UvrB subunits and scans DNA for abnormalities	LHK_01605	*L. nitroferrum*	82.89	0
	*uvrB*	Protein UvrB	Causes local melting of the DNA helix, probes one DNA strand for the presence of a lesion	LHK_00960	*L. nitroferrum*	82.18	0
	*uvrC*	Protein UvrC	Incises 5' and 3' sides of lesion	LHK_02627	*L. nitroferrum*	71	0
Transcription coupled repair factors							
DNA-directed RNA polymerase (RNAP) complex	*rpoB*	RNAP subunit beta	Subunit of DNA-dependent RNA polymerase	LHK_00246	*L. nitroferrum*	85.26	0
	*rpoC*	RNAP subunit beta	Subunit of DNA-dependent RNA polymerase	LHK_00247	*C. violaceum*	87.09	0
	*rpoA*	RNAP subunit alpha	Subunit of DNA-dependent RNA polymerase	LHK_00279	*L. nitroferrum*	90.83	1.00E-171
	*rpoE*	RNAP delta factor	Participates in initiation and recycling phases of transcription	LHK_01458	*L. nitroferrum*	63.82	7.00E-54
	*rpoZ*	RNAP omega subunit	Promotes RNA polymerase assembly	LHK_00457	*Methylobacillus flagellatus*	73.91	1.00E-21
Transcription-repair coupling factor (TRCF)	*mfd*	TRCF	Recognizes RNAP-DNA-RNA complex blocked at template strand lesion, replaces RNAP, releases truncated transcript and recruits UvrABC repair system	LHK_00629	*L. nitroferrum*	73.95	0
Mismatch excision repair							
Mismatch and loop recognition factors	*mutS*	DNA mismatch repair protein MutS	Mismatch recognition	LHK_00373	*C. violaceum*	67.73	0
Molecular matchmarker	*mutL*	DNA mismatch repair protein MutL	Promotes formation of a stable complex between two or more DNA-binding proteins	LHK_01012	*C. violaceum*	55.51	0
DNA exonucleases	*xseA*	Exodeoxyribo-nuclease 7 large subunit	Bidirectionally degrades single-stranded DNA	LHK_01101	*C. violaceum*	59.51	4.00E-125
	*xseB*	Exodeoxyribo-nuclease 7 small subunit	Bidirectionally degrades single-stranded DNA	LHK_02322	*C. violaceum*	65.28	6.00E-20
DNA polymerase III holoenzyme	*dnaE*	DNA polymerase III subunit alpha	Subunit of DNA polymerase	LHK_01389	*L. nitroferrum*	74.13	0
	*dnaN*	DNA polymerase III subunit beta	Subunit of DNA polymerase, initiates replication	LHK_03241	*L. nitroferrum*	72.5	3.00E-131
	*holC*	DNA polymerase III subunit chi	Subunit of DNA polymerase	LHK_01415	*C. violaceum*	50	2.00E-27
	*holA*	DNA polymerase III subunit delta	Subunit of DNA polymerase, interacts with gamma subunit to transfer beta subunit on DNA	LHK_00117	*C. violaceum*	67.28	7.00E-79
	*holB*	DNA polymerase III subunit delta	Subunit of DNA polymerase	LHK_02696	*L. nitroferrum*	57.36	3.00E-75
	*dnaQ*	DNA polymerase III subunit epsilon	Subunit of DNA polymerase, a 3'-5' exonuclease posseses proofreading function	LHK_00881	*C. violaceum*	71.74	6.00E-85
				LHK_01009	*C. violaceum*	62.7	4.00E-60
				LHK_02526	*C. violaceum*	51.52	3.00E-105
	*dnaX*	DNA polymerase III subunits gamma and tau	Subunits of DNA polymerase, tau subunit serves as scaffold in dimerization of the core complex while gamma subunit interacts with delta subunit to transfer beta subunit on DNA	LHK_00963	*C. violaceum*	82.17	2.00E-154
Other MMR factors	*dam*	DNA adenine methylase	Methylates DNA sequence GATC and protects DNA from cleavage by restriction endonuclease	LHK_01749	*C. violaceum*	83.92	8.00E-131
				LHK_02602	*C. violaceum*	75	9.00E-113
				LHK_00398	*C. violaceum*	75	9.00E-113
	*vsr*	Very short patch repair protein	Endonuclease, nicks double-stranded DNA	LHK_03243	*Limnobacter *sp. MED105	61.38	5.00E-48

#### Base excision repair

*L. hongkongensis *is exposed to reactive oxygen species generated during normal cellular metabolism, as well as from oxidative bursts from its host. One of the most important protective defense mechanisms against such DNA damage is the base excision repair (BER) pathway, which recognizes a wide range of DNA lesions. This includes the most frequently encountered form of oxidative DNA damage: production of 7, 8-dihydro-8-oxo-2'-deoxyguanosine (8oxodG) which can lead to ambiguous base pairing (either A or C) during DNA replication. The BER pathway is carried out by two types of enzymes: glycosylases and AP-endonucleases. Glycosylases excise the damaged base from the sugar phosphate backbone, leaving abasic (AP) sites, and endonucleases incise the 5' or 3' phosphodiester from the AP site to generate a nucleotide gap. There are eight glycosylases and endonucelases in the *L. hongkongensis *genome. Among the glycosylases, the uracil DNA glycocosylase (UNG) is the most well characterized enzyme found in various bacteria and eukaryotes. It is responsible for the excision of uracil residues from DNA which can arise as a result of misincorporation of dUMP residues by DNA polymerase or due to cytosine deamination. Similar to *C. violaceum *[15], the most closely related bacterial species of the *Neisseriaceae *family with complete genome sequence available, the *L. hongkongensis *genome contains two copies of UNG (Table 1). The complete 8oxodG system (GO system) is also present, which involves MutM/FPG, MutT and MutY, which act together to protect the bacterium against the effects of 8oxodG in *E. coli *[16]. MutM or FPG is formamidopyrimidine DNA glycosylase that recognizes oxidized purines such as 8oxodG and imidzole ring-opened purines; while MutY is an atypical glycosylase which removes adenine from DNA when it is mispaired with 8oxodG, preventing GC to TA transversions [17]. In *N. meningitidis*, it has been shown that MutY has a prominent role in DNA repair, with *mutY *mutants exhibiting high spontaneous mutation rates [14].

#### Nucleotide excision repair

Nucleotide excision repair (NER) involves a group of highly conserved proteins and repairs bulky lesions caused by exogenous damage such as UV light that generate a large helical distortion [18,19]. NER is carried out by the UvrABC complex in *E. coli*, which excises a 24- to 32-bp DNA fragment that contains the damaged lesion [20]. A functional NER pathway has also been demonstrated in *N. gonorrhoeae *[21]. Similar to *N. gonorrhoeae, N. meningitidis and C. violaceum *[14,15,21], homologues of all enzymes in this pathway are present in the *L. hongkongensis *genome (Table 1).

#### Mismatch repair

**Figure 1:**
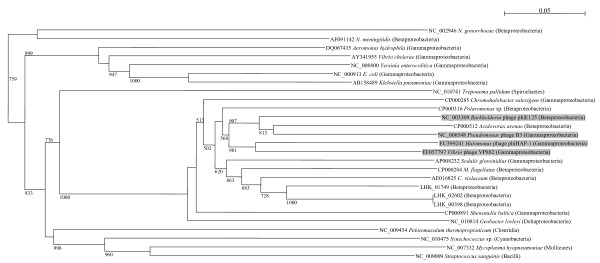
**Phylogenetic tree showing the relationships of the three copies of Dam methylases from *L. hongkongensis *(LHK_01749, LHK_02602 and LHK_00398) to those from other bacteria**. The unrooted tree was constructed by neighbor-joining method using Kimura's two-parameter correction, with bootstrap values calculated from 1000 trees. The scale bar indicates the estimated number of substitutions per 20 bases. Bacterial names and accession numbers are given as cited in the GenBank database. Phylum or class is indicated in parentheses. Genes identified in bacteriophages are highlighted in grey.

The mismatch repair (MMR) system recognizes and removes single-base mismatches as well as small nucleotide insertions or deletions (forming small loops) that result from errors during replication. In *E. coli*, MMR is carried out by a number of enzymes working at a sequential manner: MutS recognizes the mismatch; MutL is recruited and binds as a dimer; the bound MutS-MutL complex in turn recruits the MutH endonuclease; MutH nicks the nascent DNA strand, distinguishing it from the parental strand by its under-methylation of GATC sequences; MutU (also known as UvrD) and other exonucleases (such as RecJ or ExoI) mediate the removal of up to 1000 bases (upstream or downstream) of the strand that contain the lesion [22,23]. This strand is then repaired by the actions of DNA polymerase I. Similar to *N. meningitidis *and *C. violaceum *[14,15], the *L. hongkongensis *genome contains the most important enzymes of the MMR pathway except that *mutH *is absent, suggesting that this gene has been lost in related bacterial lineages (Table 1). In *N. meningitidis*, it has been shown that *mutS *mutants had a significantly increased frequency of phase variation and moderate increases in the rate of missense mutations [24]. However, other mechanisms are likely involved in determining meningococcal mutability. Further studies are required to investigate if MutH function is not required or another protein carries out the MutH strand-specificity function in these bacteria of the *Neisseriaceae *family. In contrast to *C. violaceum, N. meningitidis and N. gonorrhoeae *which possess only one copy of the Dam protein, which is responsible for DNA methylation, the *L. hongkongensis *genome contains three copies of *dam*. These three Dam homologues are phylogenetically most closely related to the Dam of *C. violaceum*, with two of the three copies having identical nucleotide sequences encoded on two highly similar prophages (Figure 1). It has previously been reported that the Dam methylase from *C. violaceum *has high similarity to a bacteriophage Dam homologue, suggesting acquisition via a horizontal transfer event [15]. Although our analysis shows that the Dam proteins from *L. hongkongensis *and *C. violaceum *are only distantly related to homologues found in other bacteriophages, the phylogenetic clustering of enzymes from different classes of bacteria supports that this enzyme is frequently horizontally transferred between bacteria (Figure 1).

#### Recombinational repair

Recombinational repair is activated in response to double-strand breaks (DSBs) in DNA which can lead to broken chromosomes and cell death. Such damage is repaired by homologous recombination in a process known as double-strand break repair (DSBR); which involves initiation, strand pairing and exchange, branch migration and branch resolution. Similar to the pathogenic *Neisseria *species and *C. violaceum *[15,25,26], the *L. hongkongensis *genome possesses all the important genes in this pathway, including the *recA *gene universally found in bacteria (Table 2). RecA has an important role in pathogenic *Neisseria *species, being involved in repeat-associated events, including those associated with pilus antigenic variation and transformation in *N. meningitidis *[25]. The *L. hongkongensis *genome contains two pathways for repair initiation (RecBCD and RecFOR), and two pathways for branch migration and resolution (RuvABC and RecG). In addition to recombination repair, the RecBCD and RecN are also involved in recombination during transformation, and RecO, RecQ and RecJ in antigenic variation in *N. gonorrhoeae *[25,27]. However, it remains to be seen if these components possess similar function in related species including *L. hongkongensis*.

**Table 2:** Homologous recombination proteins in *L. hongkongensis *and their closest homologues

Repair pathways/Types of enzymes	Gene	Protein	Function of protein encoded	CDS	Closest match organism	Amino acid Identity (%)	Best E-value
Initiation							
RecBCD pathway	*recB*	Exodeoxyribonuclease V beta chain	Catalyzes unwinding of double-stranded DNA and cleavage of single-stranded DNA, stimulates local genetic recombination	LHK_01202	*Pseudomonas entomophila*	45.33	0
	*recC*	Exodeoxyribonuclease V gamma chain	ATP-dependent exonuclease and helicase, DNA-dependent ATPase and ATP-stimulated endonuclease	LHK_01203	*Pseudomonas aeruginosa*	48.11	0
	*recD*	Exodeoxyribonuclease V alpha chain	ATP-dependent exonuclease, ATPase and ATP-stimulated endonuclease	LHK_01201	*Pseudomonas putida*	50.47	1.00E-126
RecFOR pathway	*recF*	DNA replication and repair protein RecF	DNA metabolism, DNA replication and normal SOS inducibility	LHK_01798	*Bordetella petrii*	43.67	4.00E-91
	*recO*	DNA repair protein RecO	Acts with RecF and RecR	LHK_01467	*L. nitroferrum*	50.41	6.00E-43
	*recR*	Recombination protein RecR	Acts with RecF and RecO	LHK_00965	*L. nitroferrum*	70.71	5.00E-79
	*recQ*	ATP-dependent DNA helicase RecQ	Helicase involved in the RecFOR recombination pathway	LHK_02771	*C. violaceum*	68.49	0
Branch migration and resolution	*recG*	ATP-dependent DNA helicase RecG	Catalyzes branch migration in processing Holliday junction intermediates to mature products. Unwinds DNA with a 3' to 5' polarity	LHK_02776	*L. nitroferrum*	71.3	0
	*ruvA*	Holiday junction ATP-dependent DNA helicase RuvA	Forms complex with RuvB, RuvAB is a helicase that mediates Holliday junction migration by localized denaturation and reannealing	LHK_03111	*C. violaceum*	59.7	1.00E-54
	*ruvB*	Holiday junction ATP-dependent DNA helicase RuvB	Possesses weak ATPase activity, stimulated by the RuvA protein in the presence of DNA. Forms complex with RuvA	LHK_00086	*L. nitroferrum*	92.35	8.00E-165
	*ruvC*	Crossover junction endodeoxyribonuclease RuvC	Resolves Holliday junction intermediates in recombination, cleaves cruciform structure in supercoiled DNA	LHK_03190	*L. nitroferrum*	79.89	6.00E-58
Other recombination repair related proteins	*priA*	Primosomal protein N'	Replication restart protein, catalyzes reactivation of replication forks that have stalled at sites of DNA damage	LHK_02821	*L. nitroferrum*	58.37	0
	*radA*	DNA repair and recombination protein RadA	Binds and assembles on single-stranded DNA, promotes DNA strand exchange between homologous DNA molecules	LHK_02039	*L. nitroferrum*	79.42	0
	*rusA*	Crossover junction endodeoxyribonuclease RusA	Resolves Holliday junction intermediates made during homologous genetic recombination and DNA repair	LHK_01785	*Ralstonia eutropha*	62.04	3.00E-40
	*rdgC*	Recombination-associated protein RdgC	Inhibits RecA promoted DNA strand exchange, ATPase activity, and RecA-dependent LexA cleavage, a potential negative regulator of RecA	LHK_00720	*L. nitroferrum*	58.92	3.00E-92
	*recX*	Regulatory protein RecX	Inhibits RecA recombinase and coprotease activities	LHK_00794	*Burkholderia phymatum*	52.45	8E-25
	*yqgF*	Putative Holliday junction resolvase	Nuclease resolves Holliday junction intermediates	LHK_02882	*L. nitroferrum*	66.67	8E-46
	*bet*	Single-stranded DNA annealing protein	Mediates annealing of (partially) single-stranded regions of DNA containing regions of complementary sequence	LHK_01498	*Providencia rettgeri*	69	9E-74
	*exo*	Alkaline exonuclease	Single-stranded DNA exonuclease that digests double-stranded DNA ends with 5'- to 3'-polarity to generate long 3'-ssDNA ends	LHK_01497	*Klebsiella pneumoniae *subsp. *rhinoscleromatis*	70	7E-76

Interestingly, homologues of the Bet and Exo recombinational repair proteins from bacteriophage lambda are present within a probable 11kb defective prophage region on the *L. hongkongensis *chromosome. Bet is a single-stranded DNA annealing protein (SSAP, sometimes also referred to as a synaptase), and Exo is a single-stranded DNA alkaline exonuclease with 5'- to 3'-polarity [28]. The *bet *and *exo *genes are positioned immediately adjacent to one another along with an additional copy of a single-stranded DNA binding protein of phage origin (*ssb2*, LHK_01496), which is homologous to, but distinct from, the presumed major functioning *ssb *of neisserial origin (LHK_01479). Such arrangements of phage-related DNA recombination proteins are commonly found in bacteria [29], acquired presumably by phage integration followed by subsequent genetic rearrangement. If actively transcribed, functional pairs of Exo and Bet proteins will promote DNA recombination events analogous to those mediated by the RecA/RecBCD/RecFOR pathways, and would be expected to increase the rates of gene/genome rearrangements [28]. The Bet and Exo proteins may also function synergistically with RecA. The transcriptional status of the genes within this presumed defective prophage region remain to be established.

It has previously been noted that low-GC Gram positive species tend to possess RecT SSAPs rather than Bet-family proteins [29], although this relationship has not been re-examined more recently. The LHK_01498 gene is the only *bet *homologue present in the *Neisseriaceae*. However, there is a (functionally-equivalent) *recT *homologue present in *Kingella oralis *ATCC51147 (the only recT-family recombinase present in the *Neisseriaceae*) which does appear to have partnering exonuclease. Due to likely (partial) genetic reassortment in a phage host prior to incorporation into the *L. hongkongensis *genome, the *bet*, *exo *and *ssb2 *genes have apparently unrelated phylogenies (data not shown). The 162aa Ssb2 protein homologue is 69% identical to the presumed functional Ssb protein within the cell (175aa), but protein alignment reveals that it is lacking a stretch of ca. 25 amino acids near the C-terminus (data not shown). Interestingly, structural studies on the *E. coli *Ssb-DNA complex have shown that this unstructured region loops out from the SsB tetramer [30]. This region is not involved in DNA binding, but is thought to be responsible for interacting with the DNA primase and clamp loader proteins [31]. This suggests that the Ssb and Ssb2 proteins are designed to work with quite different replication or DNA repair protein systems.

#### SOS Response

The SOS response is activated when replication is blocked by DNA damage. The pathway is responsible for activation of a variety of physiological responses, including cell cycle inhibition and various DNA repair pathways. In *E. coli*, the SOS response involves more than 40 genes which are induced when there is a large amount of DNA damage, allowing increased repair and restoration of replication [32]. The pathway is controlled by a dual-component system, with RecA being the activator and LexA the repressor. The RecA protein forms a complex with single-stranded DNA, which leads to cleavage of LexA repressor and expression of the SOS regulon. Although genes related to SOS response, including *dinB*, *dinG*, *umu-D *and *dnaA*, could be identified, the *lexA *is absent in *L. hongkongensis *genome, a phenomenon also observed in *C. violaceum*, *N. meningitidis *and *N. gonorrhoeae *[14,15,33] (Table 3). This suggests that the *lexA *gene is lost in the common ancestor of these bacteria during evolution. Moreover, the *recA*, *uvrA *and *uvrB *genes of *N. gonorrhoeae *are known to lack the characteristic *lexA*-binding site or SOS boxes, the general hallmarks of an active SOS response. In fact, it is been experimentally confirmed that a functional SOS response is absent in *N. gonorrhoeae *[34]. Similarly, SOS boxes cannot be identified in the homologues of SOS-inducible genes in *N. meningitidis *[14,35,12], suggesting that the SOS response may also be absent in related bacteria of the same family. Similar to the two *Neisseria *species and *C. violaceum *[15], SOS boxes are also absent in the SOS-related genes in *L. hongkongensis*. Further studies are required to determine if SOS response is constitutive or absent in this group of bacteria.

**Table 3:** Other proteins involved in DNA repair

Repair pathways/Types of enzymes	Gene	Protein	Function of protein encoded	CDS	Closest match organism	Amino acid Identity (%)	Best E-value
TLS (translesion DNA synthesis) factors							
Y-family DNA polymerases	*dinB*	DNA Polymerase IV	Poorly processive, error-prone DNA polymerase involves in translesional DNA synthesis	LHK_01833	*L. nitroferrum*	69.32	2.00E-128
	*umuD*	Protein UmuD	Essential for induced (or SOS) mutagenesis, modifies DNA replication machinery to allow bypass synthesis across a damaged template	LHK_01580	*Legionella pneumophila *subsp. *pneumophila*	48.65	9.00E-32
Other SOS response factors	*dinG*	Probable ATP-dependent helicase DinG	Damage-inducible helicase, unwinds DNA duplex with a 5'-3'-polarity	LHK_02134	*L. nitroferrum*	64.79	0
	*dnaA*	Chromosomal replication initiator protein DnaA	Initiates and regulates chromosomal replication	LHK_03240	*L. nitroferrum*	76.72	0
Modulation of nucleotide pools	*dut*	dUTPase	Produces dUMP, immediate precursor of thymidine nucleotides and decreases intracellular concentration of dUTP	LHK_01910	*L. nitroferrum*	78.45	1.00E-46
	*nrdA*	Ribonucleoside-diphosphate reductase 1 subunit alpha	Catalyzes biosynthesis of deoxyribo-nucleotides from the corresponding ribonucleotides	LHK_01803	*L. nitroferrum*	71	0
	*nrdB*	Ribonucleoside-diphosphate reductase 1 subunit beta	Catalyzes biosynthesis of deoxyribo-nucleotides from the corresponding ribonucleotides	LHK_01801	*L. nitroferrum*	83.1	5.00E-177
	*nrdE*	Ribonucleoside-diphosphate reductase 2 subunit alpha	Catalyzes biosynthesis of deoxyribo-nucleotides from the corresponding ribonucleotides	LHK_01596	*L. nitroferrum*	79.73	0
	*mutT*	Mutator MutT protein	Removes oxidatively damaged guanine from DNA and the nucleotide pool, degrades 8-oxo-dGTP to monophosphate	LHK_02262	*C. violaceum*	60.12	2.00E-56
Other factors involved in DNA repair	*ligA*	DNA ligase	Catalyzes phosphodiester linkages between 5'-phosphoryl and 3'-hydroxyl groups in double-stranded DNA, essential for DNA replication and repair	LHK_02877	*Cupriavidus taiwanensis*	65.83	0
	*recJ*	Single-stranded-DNA-specific exonuclease RecJ	Single-stranded-DNA-specific exonuclease required for many recombinational events	LHK_02397	*L. nitroferrum*	71.58	0
	*polA*	DNA polymerase I	DNA polymerase exhibits 3' to 5' and 5' to 3' exonuclease activity	LHK_02983	*C. violaceum*	68.03	0
	*ssb*	Single-stranded DNA binding protein, SSB	Forms homotetramer and binds single-stranded DNA to protect susceptible ssDNA from nucleolytic digestion and prevents secondary-structure formation	LHK_01479	*M. flagellatus*	82.24	4.00E-46
	*ssb2*	Single-stranded DNA binding protein	Forms a homotetramer and binds single-stranded DNA to protect susceptible ssDNA from nucleolytic digestion and prevents secondary-structure formation	LHK_01496	*L. hongkongensis *HLHK9 (SSB protein)	68	3E-57
	*recA*	Protein RecA	Catalyzes ATP-dependent uptake of single-stranded DNA by duplex DNA, and hybridization of homologous single-stranded DNA	LHK_00793	*L. nitroferrum*	86.75	4.00E-137
	*recN*	DNA repair protein RecN	Coordinates alignment of broken segments with intact duplexes to facilitate recombination	LHK_01210	*C. violaceum*	62.43	5.00E-159
	*uvrD*	DNA helicase II	ATPase and helicase involves in post-incision events of nucleotide excision repair and methyl-directed mismatch repair	LHK_00065	*C. violaceum*	65.68	0
	*rep*	ATP-dependent DNA helicase Rep	Helicase and ATPase involves in DNA replication, binds to single-stranded DNA, initiates unwinding at a nick	LHK_00318	*L. nitroferrum*	72.86	0

### DNA replication

Bacterial DNA replication mechanisms are responsible for the accurate duplication of genetic material during cell division. The whole process involves the interplay of many different proteins with a variety of functions. A total of 36 coding sequences (CDSs) potentially involved in DNA replication are present in the *L. hongkongensis *genome, including 12 initiation factors, 11 elongation factors, 2 termination factors and 5 topoisomerases (Table 4). Since many of these proteins are essential to the bacterial cell and therefore preserved during bacterial evolution, they are often highly conserved among phylogenetically closely related bacteria.

**Table 4:** Replication proteins and their closest homologues

Types of enzymes	Gene	Protein	Function of protein encoded	CDS	Closest match organism	Amino acid identity (%)	Best E-value
Initiation factors	*hupB1*	DNA-binding protein hu-beta	Beta chain of heterodimeric histone-like DNA-binding protein, wraps DNA to stabilize and prevent denaturation under extreme environmental conditions	LHK_02345	*C. violaceum*	79.78	2.00E-33
	*hupB2*	DNA-binding protein hu-beta	Beta chain of heterodimeric histone-like DNA-binding protein, wraps DNA to stabilize and prevent denaturation under extreme environmental conditions	LHK_02180	*C. violaceum*	46.59	1.00E-14
	*ihfA/himA*	Integration host factor subunit alpha	One of the two subunits of integration host factor, a specific DNA-binding protein	LHK_02751	*L. nitroferrum*	91.84	8.00E-46
	*ihfB/himD*	Integration host factor subunit beta	One of the two subunits of integration host factor, a specific DNA-binding protein	LHK_00870	*L. nitroferrum*	82.35	2.00E-39
	*dnaA*	Chromosomal replication initiator protein DnaA	Initiates and regulates chromosomal replication	LHK_03240	*L. nitroferrum*	76.72	0
	*dnaB*	Replicative DNA helicase	Initiation and elongation, DNA-dependent ATPase	LHK_01738	*C. violaceum*	76.48	0
				LHK_01506	*N. gonorrhoeae*	40.73	3.00E-76
	*dnaG*	DNA primase	Polymerase synthesizes small RNA primers for the Okazaki fragments on both template strands at replication forks	LHK_00463	*C. violaceum*	65.42	2.00E-155
	*ssb*	Single-stranded DNA binding protein, Ssb	Forms homotetramer and binds single-stranded DNA to protect susceptible ssDNA from nucleolytic digestion and prevents secondary-structure formation	LHK_01479	*M. flagellatus*	82.24	4.00E-46
	*ssb2*	Single-stranded DNA binding protein	Forms a homotetramer and binds single-stranded DNA to protect susceptible ssDNA from nucleolytic digestion and prevents secondary-structure formation	LHK_01496	*L. hongkongensis *HLHK9 (Ssb protein)	68	3E-57
	*fis*	DNA-binding protein Fis	Nucleoid-associated protein	LHK_03207	*C. violaceum*	73.68	1.00E-25
	*hvrA*	H-NS like protein	Binds tightly to dsDNA, increases thermal stability and inhibits transcription	LHK_00853	*C. violaceum*	58.82	4.00E-28
				LHK_00959	*C. violaceum*	53.47	2.00E-18
	*iciA*	Chromosome initiation inhibitor	In vitro inhibitor of chromosomal replication initiation	LHK_00797	*Acinetobacter baumannii*	43.24	1.00E-63
Elongation factors	*dnaE*	DNA polymerase III subunit alpha	Subunit of DNA polymerase	LHK_01389	*L. nitroferrum*	74.13	0
	*dnaN*	DNA polymerase III subunit beta	Subunit of DNA polymerase, initiates replication	LHK_03241	*L. nitroferrum*	72.5	3.00E-131
	*holC*	DNA polymerase III subunit chi	Subunit of DNA polymerase	LHK_01415	*C. violaceum*	50	2.00E-27
	*holA*	DNA polymerase III subunit delta	Subunit of DNA polymerase, interacts with gamma subunit to transfer beta subunit on DNA	LHK_00117	*C. violaceum*	67.28	7.00E-79
	*holB*	DNA polymerase III subunit delta'	Subunit of DNA polymerase	LHK_02696	*L. nitroferrum*	57.36	3.00E-75
	*dnaQ*	DNA polymerse III subunit epsilon	Subunit of DNA polymerase, a 3'-5' exonuclease possesses proofreading function	LHK_00881	*C. violaceum*	71.74	6.00E-85
				LHK_01009	*C. violaceum*	62.7	4.00E-60
				LHK_02526	*C. violaceum*	51.52	3.00E-105
	*dnaX*	DNA polymerse III subunits gamma and tau	Subunits of DNA polymerase, tau subunit serves as scaffold in dimerization of the core complex while gamma subunit interacts with delta subunit to transfer beta subunit on DNA	LHK_00963	*C. violaceum*	82.17	2.00E-154
	*rnhA*	Ribonuclease HI	Endonuclease degrades RNA of RNA-DNA hybrids, specifies the origin of replication by suppressing initiation at origins other than the oriC locus, removes RNA primers from the Okazaki fragments of lagging strands	LHK_00880	*L. nitroferrum*	77.3	2.00E-59
	*rnhB*	Ribonuclease HII	Endonuclease degrades RNA of RNA-DNA hybrids	LHK_00722	*L. nitroferrum*	71.88	1.00E-68
	*polA*	DNA polymerase I	DNA polymerase exhibits 3' to 5' and 5' to 3' exonuclease activity	LHK_02983	*C. violaceum*	68.03	0
	*ligA*	DNA ligase	Catalyzes phosphodiester linkages between 5'-phosphoryl and 3'-hydroxyl groups in double-stranded DNA, essential for DNA replication and repair	LHK_02877	*C. taiwanensis*	65.83	0
Termination factors	*dam*	DNA adenine methylase	Methylates DNA within the sequence GATC and protects the DNA from cleavage by restriction endonuclease	LHK_01749	*C. violaceum*	83.92	8.00E-131
				LHK_02602	*C. violaceum*	75	9.00E-113
				LHK_00398	*C. violaceum*	75	9.00E-113
	*hda*	DnaA-homolog protein hda	Mediates interactions of DnaA with beta subunit sliding clamp, controls initiation of DNA replication by inhibiting reinitiation of replication	LHK_00510	*C. violaceum*	66.82	1.00E-77
Topoisomerases	*gyrA*	DNA gyrase subunit A	Negatively supercoils closed circular double-stranded DNA, catalyzes interconversion of topological isomers of double-stranded DNA rings, including catenanes and knotted rings. Consists of subunit A and B. Responsible for DNA breakage and rejoining, forms A2B2 tetramer	LHK_01836	*L. nitroferrum*	82.09	0
	*gyrB*	DNA gyrase subunit B	Negatively supercoils closed circular double-stranded DNA, catalyzes interconversion of topological isomers of double-stranded DNA rings, including catenanes and knotted rings. Consists of subunit A and B. Catalyzes ATP hydrolysis, forms A2B2 tetramer	LHK_03242	*L. nitroferrum*	83.06	0
	*parC*	DNA topoisomerse 4 subunit A	Essential for chromosome segregation, relaxation of supercoiled DNA. Performs decatenation during replication of circular DNA molecule. Composed of subunits ParC and ParE	LHK_00093	*L. nitroferrum*	74.47	0
	*parE*	DNA topoisomerase 4 subunit B	Essential for chromosome segregation, relaxation of supercoiled DNA. Performs decatenation during replication of circular DNA molecule. Composed of subunits ParC and ParE	LHK_00606	*L. nitroferrum*	82.01	0
	*topA*	DNA topoisomerase 1	Conversion of one DNA topological isomer to another	LHK_03143	*C. violaceum*	80.13	0

#### Replication initiation

In *L. hongkongensis*, there is an *oriC *containing eight 9-bp repeat elements known as DnaA boxes, which are potential binding sites for the initiator protein DnaA. The DnaA in *L. hongkongensis *is highly conserved when compared to those in closely related bacteria, with 76.7% amino acid identity with the homologue from *Lutiella nitroferrum*. The four domains of DnaA previously identified to possess distinct functions are also present [36]. As DnaA assembles with *oriC *to form a large nucleoprotein complex, the DNA melts to generate single DNA strands necessary for the binding of a helicase, DnaB, and the replisomal machinery [36]. Although bacteria do not possess histones, their genomes are arranged in tightly compacted arrangements known as nucleoids, which are important for maintaining an optimal DNA topology for replication initiation.

Six nucleoid-associated proteins, also referred to as histone-like factors, were identified in the *L. hongkongensis *genome, including two HU-beta proteins, one HN-S protein, two integration host factors (IHF) and one factor for inversion stimulation (Fis). HU-beta and HN-S proteins bind DNA non-specifically and contribute to the global condensation of bacterial chromosomes [37]. IHF and Fis recognize specific DNA sequences and assist in organizing supercoiled domains [36,38]. Earlier studies have shown that IHF stimulates DnaA-mediated unwinding of *oriC*, whereas Fis inhibits DUE melting [39,40].

#### Replication regulation

The *L. hongkongensis *genome contains three copies of *dam *and one copy of the *hda *gene which are likely involved in the regulation of the replication process. Dam is an adenine methyltransferase responsible for the methylation of GATC sites of the *oriC *in *E. coli *which is important for origin sequestration, thus preventing re-initiation. Hda, a homologue of DnaA, is involved in the regulatory inactivation of DnaA (RIDA), which directly stimulates ATP hydrolysis by DnaA after the initiator melts the DUE [36]. It has been shown that *hda*-deficient cells display an over-initiation phenotype in *E. coli *[41,42].

### Control of gene expression

As for other bacteria, the principal mechanism for control of gene expression is through regulation of the amount of mRNA produced from the corresponding gene. This is primarily determined by the affinity of RNA polymerase for the promoter. In *L. hongkongensis*, this is exemplified by the difference in mRNA levels of argB-20 and argB-37 at different temperatures, resulting in different amounts of the two enzymes, N-acetyl-L-glutamate kinase (NAGK)-20 and NAGK-37 respectively [43]. Genes that encode proteins which control basal transcription, including the five-subunit RNA polymerase core enzyme (α_2_ββ'ω) and σ-factors for binding specifically to different classes of promoters and hence selective expression of different groups of genes, are present in the *L. hongkongensis* genome. The primary σ-factor, σ^70^, is responsible for recognizing the promoters for transcription of most of the housekeeping genes. Furthermore, the *L. hongkongensis* genome contains other alternative σ-factors, including σ^28 ^(FliA), σ^32 ^(RpoH), σ^38 ^(RpoS), σ^24 ^(RpoE) and σ^54 ^(RpoN), which allow it to bring about global changes in gene expression in response to different environmental stresses (Table 5). The types of alternative σ-factors in *L. hongkongensis* are the same as those in *C. violaceum*, except that there are two copies of σ^28 ^(the flagellar σ-factor) in the *C. violaceum* genome but only one copy of σ^28 ^in the *L. hongkongensis* genome. In the genomes of *N. gonorrhoeae *and *N. meningitidis*, no σ^28 ^and σ^38 ^(the starvation/stationary phase σ-factor) are observed. In addition to RNA polymerase and the σ-factors, the *L. hongkongensis* genome also encodes transcriptional activators and repressors, which belong to a variety of families of transcription factors. These transcription factors bind to sites near the target promoter and stimulate or repress the activity of the corresponding σ-RNA polymerase holoenzyme. In the *L. hongkongensis* genome, 109 coding sequences (CDSs) that encode putative transcription factors were identified (Table 6). Among the 46 families of bacterial transcription factors, *L. hongkongensis* contains genes that encode putative transcription factors in 22 of them. The largest groups belong to the LysR families. In most of the families, the number of genes in the *L. hongkongensis* genome that encode putative transcription factors in that family is in between that of *C. violaceum* and the *Neisseria* species (Table 7). This is in line with the ability of *C. violaceum* to survive in a wide range of environments and the fastidious growth requirements and limited host range of *N. gonorrhoeae* and *N. meningitidis*. One of the exceptions is that *L. hongkongensis* possesses three CDSs that encode putative transcription factors of the cold shock family, more than those in the genomes of *C. violaceum*, *N. gonorrhoeae* and *N. meningitidis*. This may be related to the adaptability of *L. hongkongensis* to environments of low temperatures, such as those of freshwater fish and frogs.

**Table 5:** CDSs related to transcription in *L. hongkongensis, N. meningitidis, N. gonorrhoeae *and *C. violaceum*

Product	Gene	*L. hongkongensis *HLHK 9	*N. meningitidis *MC58	*N. gonorrhoeae *FA1090	*C. violaceum *ATCC12472
ATP-dependent helicase	*hrpA*	+	+	+	+
ATP-dependent RNA helicase	*rhlE*	+	+	+	+
DNA-directed RNA polymerase (alpha subunit)	*rpoA*	+	+	+	+
DNA-directed RNA polymerase (beta subunit)	*rpoB*	+	+	+	+
DNA-directed RNA polymerase (beta subunit)	*rpoC*	+	+	+	+
DNA-directed RNA polymerase (omega subunit)	*rpoZ*	+	+	+	+
RNA helicase	*dbpA*	-	-	-	+
Sigma factor 32	*rpoH*	+	+	+	+
Sigma factor 38	*rpoS*	+	-	-	+
Sigma factor A (sigma 70)	*rpoD*	+	+	+	+
Sigma factor E (sigma 24)	*rpoE*	+	+	+	+
Sigma factor for flagellar operon	*fliA*	+	-	-	+^a^
Sigma factor N (sigma 54)	*rpoN*	+	+	+	+
Transcription elongation factor GreA	*greA*	+	+	+	+
Transcription elongation factor GreB	*greB*	+	+	+	+
Transcription termination factor Rho	*rho*	+	+	+	+
N utilization substance protein A	*nusA*	+	+	+	+
N utilization substance protein B	*nusB*	+	+	+	+
Transcription anti-termination protein NusG	*nusG*	+	+	+	+

^a^Two copies of the gene are present

**Table 6:** Families of transcription factors (TFs) in *L. hongkongensis*

Family	Number of TFs	Family	Number of TFs
LysR	25	PadR	0
AraC/XylS	9	RpiR	0
OmpR	9	ArgR	0
NtrC/Fis	8	DtxR	0
TetR	8	LexA	0
CRO/CI/Xre	8	TrmB	0
LuxR	7	PenR/BlaI/MecI	0
GntR	6	SfsA	0
ArsR	4	CopG/RepA	0
MarR	4	ModE	0
Cold shock domain	3	PaiB	0
MerR	3	CtsR	0
AsnC	2	CodY	0
CRP-FNR	2	TrpR	0
DeoR	2	MtlR	0
Fur	2	ROS/MUCR	0
BolA/YrbA	2	MetJ	0
IclR	1	GutM	0
Rrf2	1	Crl	0
LytTR	1	ComK	0
HrcA	1	FlhD	0
SirB	1	RtcR	0
LacI	0	NifT/FixU	0

**Table 7:** Distribution of transcription factors families in *L.hongkongensis*, *N. meningitidis*, *N. gonorrhoeae *and *C. violaceum*.

Transcription factor family	*L. hongkongensis *HLHK9	*C. violaceum *ATCC 12472	*N. gonorrhoeae *FA 1090	*N. meningitidis *MC58
AraC/XylS	9	25	3	3
ArsR	4	4	4	2
AsnC	2	6	2	2
Cold shock domain	3	2	1	1
CRP-FNR	2	3	1	1
DeoR	2	4	1	1
GntR	6	15	2	2
IclR	1	2	1	1
LacI	0	2	0	0
LuxR	7	12	1	1
LysR	25	67	5	6
MarR	4	18	2	3
MerR	3	8	1	1
NtrC/Fis	8	19	4	3
OmpR	9	11	1	1
TetR	8	17	2	2
CRO/CI/Xre	8	9	12	9
Fur	2	1	1	1
HrcA	1	1	0	0
SirB	1	1	1	1
Rrf2	1	2	2	2
BolA/YrbA	2	2	2	2
LytTR	1	4	0	0

### Tolerance to acid stress

*L. hongkongensis* is able to grow at pH of as low as 2 (unpublished data), and its tolerance to acid stress is much higher than that of *N. gonorrhoeae*, *N. meningitidis* and *C. violaceum*. This is in line with the recovery of *L. hongkongensis* from stool samples of patients with gastroenteritis, as it has to pass through the highly acidic environment of the stomach before reaching the intestine. Therefore, it is not surprising that *L. hongkongensis* possesses abundant mechanisms for tolerating acid stress compared to *N. gonorrhoeae*, *N. meningitidis* and *C. violaceum*.

The genome of *L. hongkongensis* contains a complete urease gene cassette and two arc gene clusters. The urease cassette contains eight CDSs encoding three structural (UreA, UreB and UreC) and five accessory proteins (UreE, UreF, UreG, UreD and UreI), whereas each arc cluster consists of four CDSs encoding the three enzymes, arginine deiminase, ornithine carbamoyltransferase and carbamate kinase, of the arginine deiminase pathway, and a membrane bound arginine-ornithine antiporter. Urease hydrolyzes urea into carbon dioxide and ammonia, whereas the arginine deiminase pathway converts L-arginine to carbon dioxide, ATP, and ammonia. The ammonia generated from both pathways raises the pH and counteracts the acid stress. A similar urease gene cassette is not present in the genomes of *N. gonorrhoeae*, *N. meningitidis* and *C. violaceum*, whereas one arc gene cluster is present in the *C. violaceum *genome, but not in that of *N. gonorrhoeae *or *N. meningitidis*.

In addition to the urease cassette and arc clusters, the *L. hongkongensis* genome also contains three CDSs that encode putative chaperones of which their transcription can also potentially be induced by acid shock. These include dnaK, mopA1 and htpG. Furthermore, other gene products may help the bacterium to survive in acidic environment or their expression can be induced by acid stress (Table 8). The functions of some of these gene products are unknown, but the survival of the respective bacteria at low pH had been shown to be affected if the corresponding gene was deleted [44-49].

**Table 8:** Other CDSs related to acid stress in *L.hongkongensis, N. meningitidis, N. gonorrhoeae *and C. violaceum.

Product	Gene	*L. hongkongensis *HLHK9	*N. meningitidis *MC58	*N. gonorrhoeae *FA1090	*C. violaceum *ATCC12472
Acid shock RNA protein	*asr*	+^a^	-	-	+
Acid-resistance protein, possible chaperone	*hdeA*	+^a^	-	-	-
Sigma factor 38	*rpoS*	+	-	-	+
Ferric uptake regulator protein	*fur*	+	+	+	+
DNA polymerase I	*polA*	+	+	+	+
*β*-ketoacyl-ACP synthases II	*fabF*	+	+	+	+
Lysine:cadaverine antiporter	*cadB*	+	-	-	+
Arginine decarboxylase	*adiA*	+	-	-	+
Ada transcriptional dual regulator	*ada*	-	-	-	+
Lysine decarboxylase	*cadA*	-	-	-	+
OmpR transcriptional dual regulator	*ompR*	-	+	+	+

^a^Two copies of the gene are present

### Tolerance to alkaline stress

*L. hongkongensis *is able to grow at pH as high as 9.0 (unpublished data). While this may be related to its ability to survive the alkaline pH in the host intestine, growth at such alkalinity is still in line with many other non-extremophilic bacteria. Adaptive mechanisms to achieve cytoplasmic pH homeostasis in bacteria include transporters and enzymes that promote proton capture and retention, production of acidic metabolites and cell surface changes [50]. Among all these mechanisms, the most widely studied ones involve the transporters.

In the genome of *L. hongkongensis*, there are four CDSs coding for putative transporters which belong to the monovalent cation/proton antiporter-2 (CPA2) family. Two of the CDSs code for putative Na^+^/H^+ ^exchangers (LHK_02296, LHK_00707) while the other two code for the putative genes *kef *(LHK_02848) and *kefB *(LHK_02018). No CDS encoding putative homologue of monovalent cation/proton antiporter-1 (CPA1) or monovalent cation/proton antiporter-3 (CPA3) has been identified. Transporters of the monovalent cation/proton antiporter (CPA) superfamily support key physiological functions of bacteria by catalyzing active efflux of Na^+ ^and/or K^+^, with respective H^+ ^influx, to maintain cytoplasmic pH homeostasis and tolerate fluctuations in osmolarity [51]. Since cytoplasmic bacterial parasites or symbionts are sheltered by the host cell, it has been postulated that their genomes encode few genes for Na^+^/H^+ ^antiporters [51]. A comparison of *L. hongkongensis*, *C. violaceum*, intracellular pathogens *N. gonorrhoeae *and *N. meningitidis*, as well as the two model bacterial organisms, *Bacillus subtilis *and *Escherichia coli*, are shown in Table 9. It can be observed that *L. hongkongensis *and *C. violaceum *have more genes predicted to encode CPA2 superfamily transporters when compared to *N. meningitidis *and *N. gonorrhoeae*.

**Table 9:** Cation/proton antiporters identified in *L. hongkongensis, N. meningitidis, N. gonorrhoeae and C. violaceum; and the model bacterial organisms, B. subtilis and E. coli*

Features	*L. hongkongensis *HLHK9	*N. meningitidis *MC58^b^	*N. gonorrhoeae *FA 1090^b^	*C. violaceum *ATCC12472	*B. subtilis *168^b^	*E. coli *K12-MG1655^b^
Genome size (Mb)^a^	3.17	2.27	2.15	4.75	4.22	4.64
Total no. of transporter proteins	442	103	96	564	298	354
No. of identified transporters per Mb genome	139	45.4	44.7	119	71.0	76.3
No. of cation/proton antiporters	6	4	4	3	6	7
Monovalent cation:proton antiporter-1 (CPA1) family	0	1	1	0	1	2
Monovalent cation:proton antiporter-2 (CPA2) family	4	1	1	3	2	3
Monovalent cation (K^+ ^or Na^+^):proton antiporter-3 (CPA3) family	0	0	0	0	1^c^	0
NhaA Na^+^:H^+ ^antiporter family	0	0	0	0	0	1
NhaB Na^+^:H^+ ^antiporter family	0	0	0	0	0	1
NhaC Na^+^:H^+ ^antiporter family	2	2	2	0	2	0
NhaD Na^+^:H^+ ^antiporter family	0	0	0	0	0	0

^a^Genome size data obtained from www.ncbi.nlm.nih.gov/projects/genome/, calculations based on data from www.membranetransport.org and with updated number of transporters in our annotation

^b^Retrieved from www.membranetransport.org

^c^Not listed on www.membranetransport.org, see Krulwich *et. al*.

In addition to the four CDSs coding for the putative transporters of the CPA2 family, two CDSs putatively coding for transporters of the NhaC Na^+^:H^+ ^antiporter (NhaC) family are also present (LHK_00646, LHK_02247) in the *L. hongkongensis *genome. Both are predicted to code for the gene *nhaC*. Nevertheless, gene sequences of the two CDSs are significantly different, indicating a possible difference in phylogenetic origin. The *nhaC *homologue in the alkaliphilic bacteria *Bacillus firmus *has been confirmed experimentally to produce NhaC, which has Na^+^/H^+ ^antiporter activity [52]. Table 9 also compares the number of identified NhaA, NhaB, NhaC and NhaD family transporters in the genomes of *L. hongkongensis *to those in *C. violaceum*, *N. meningitidis*, *N. gonorrhoeae*, *B. subtilis *and *E. coli*.

### Acquisition of and tolerance to heavy metals

To adapt to natural freshwater, *L. hongkongensis* should be able to acquire essential heavy metal ions and expel them, or their toxic counterparts, when their levels reach toxicity. Many heavy metals belong to the transition elements. Their electronic configurations provide them with an exquisite ability to form complex compounds. Metal ions such as iron(II), cobalt(II), nickel(II) and copper(II) are essential to many physiological functions, yet are toxic at high concentrations. Certain species, such as silver(I), cadmium(II) and mercury(II), however, are relatively toxic to bacteria; the toxic complexes formed by these ions preclude their physiological use by common bacteria [53].

#### Iron

Iron is required by both prokaryotes and eukaryotes for the synthesis of important proteins such as cytochromes. Bacteria employ a variety of mechanisms to acquire iron, such as siderophore-mediated uptake, metal inorganic transport systems (MIT) and ATP-binding cassette (ABC) transport systems.

No gene for siderophore production was found in the *L. hongkongensis* genome. Since heme-bound iron and iron-containing proteins may not be readily available outside of a host [54], transporter-mediated transport of ionic iron would be the probable mechanism of iron acquisition during the environmental persistence of *L. hongkongensis*. A locus coding for the periplasmic ferric iron binding protein FbpA, permease FbpB and a putative iron-transport system ATP-binding protein is present (LHK_02634-02636). Putative homologous loci, containing three similar CDSs, is present in *C. violaceum *(CV1908-1910), *N. gonorrhoeae *(NGO0215-0217) and *N. meningitidis* (NMB0632-0634). The gene coding for the putative iron-transport system ATP-binding protein in *L. hongkongensis* (LHK_02636) is probably homologous to the fbpC gene in *N. meningitidis*. The FbpABC system has been shown to be a specific ferric iron transport system with high affinity to Fe^3+ ^in *Haemophilus influenzae*[55]. In addition, two CDSs are the putative homologues of the genes coding for the high-affinity ABC transport system for ferrous iron in *E. coli* (feoABC) are present in the *L. hongkongensis* (LHK_03044-03045). The two CDSs code for the putative homologues of feoA and feoB respectively. The putative homologue of feoB is also present in *C. violaceum*. No putative homologues of feoA or feoC are found in *N. gonorrhoeae *and *N. meningitidis*.

#### Nickel

Nickel is an essential component of urease, which is implicated in the acid tolerance of *L. hongkongensis*. The CorA and HoxN systems have been proposed as an important nickel and cobalt transport system in bacteria [53]. No putative CDS coding for genes of the CorA system is present in the *L. hongkongensis* genome, yet a CDS coding for a histidine-rich glycoprotein with functional domain of the high-affinity nickel transport protein NicO was identified (LHK_02812). The NicO protein is related to the NixA of the HoxN family, which has been implicated in the urease-dependent pathogenesis of *Helicobacter pylori*[56]. A locus of four CDSs coding for dppB, dppC, dppD and dppF (LHK_00939-00942) was found. They belong to an ABC transporter subfamily and are predicted to transport dipeptides, oligopeptides and nickel. The dppA homologue (LHK_00667) is located distant from the dppBCDF locus. This is in contrast to *C. violaceum*, where the CDS coding for the putative dppA gene is contiguous to the dppBCDF locus. This separation of dppA from the dppBCDF locus, however, is not unique; it is also observed in *H. influenzae*[57]. The relatively well-characterized nickel/cobalt resistance system Cnr [58] and nickel/cobalt/cadmium resistance system Ncc [59] find no direct homologous systems in the *L. hongkongensis*; whilst the putative gene coding for a protein of similar function, in terms of sequence homology and functional domains, is traced to a CDS which encodes the putative NolG efflux pump of the resistance-nodulation-cell division superfamily (LHK_02819). In addition, two CDSs coding for an ABC transporter for cobalt (LHK_01077-01078) were found; it is uncertain whether this member of the nickel(II)-cobalt(II) uptake transporter (NiCoT) family may also transport nickel due to variations in the exact level of binding specificity [60]. Homologues of putative genes encoding dppA, dppBCDF (LHK_00667, LHK_00939-00942) and the histidine-rich glycoprotein (LHK_02812) were identified in the genome of *C. violaceum *but not published genomes of *N. gonorrhoeae *or *N. meningitidis*. No putative homologue of the locus containing genes coding for the ABC transporter for cobalt (LHK_01077-01078) was identified in *C. violaceum*, *N. gonorrhoeae *or *N. meningitidis*.

#### Cobalt

Cobalt is found in coenzyme B_12_, which is responsible for methyl group transfer and rearrangement [61,62]. Apart from the HoxN and NiCoT described, three CDSs that encode a putative ABC-type cobalt transport system (LHK_01956-01958) and one that codes for a putative magnesium and cobalt efflux protein (LHK_00289) were also found. No putative homologue of the ABC-type cobalt transport system was found in *C. violaceum*, *N. meningitidis* and *N. gonorrhoeae*. For the putative magnesium and cobalt efflux protein gene, putative homologues were found in *C. violaceum*, *N. meningitidis* and *N. gonorrhoeae *as corC.

#### Cadmium

A CDS coding for a cadmium-translocating P-type ATPase (CadA-1, LHK_00449) was found in the genome. CadA and CadA-like proteins have been implicated in the transport of various heavy metals include, but not limiting to, cadmium, cobalt, mercury, lead and zinc [53]; CadA has been shown to be responsible for the Cd^2+ ^efflux in both Gram-positive bacteria such as *Staphylococcus aureus*[63] and *Bacillus* spp. [64]; and Gram-negative bacteria such as *Ralstonia metallidurans*[65]. It may also contribute to Pb^2+ ^efflux [66]. *cadA-1 *is very similar to the *E. coli *gene *zntA*, which has been shown to be responsible for the intrinsic resistance of *E. coli* to zinc and cadmium [67]. Probable homologues and paralogues of cadA-1 and CadA-like protein coding genes are present in *C. violaceum *as zntA and copA; in *N. meningitidis* and *N. gonorrhoeae *as putative transport ATPase genes.

#### Copper

A locus of two CDSs (LHK_03034-03035) coding for a putative copper translocating ATPase and a conserved heavy metal associated domain were also found in the genome. The putative copper translocating ATPase gene has a homologue, copA, in *E. coli*; copA in *E. coli* has been shown to be important in resistance to the toxic effects of copper, and is induced by silver and copper ions [68]. Putative homologues of this copper translocating ATPase gene (LHK_03035) are also present in *C. violaceum *(copA), *N. meningitidis* and *N. gonorrhoeae*.

### Tolerance to temperature stress

*L. hongkongensis* inhabits the intestines of freshwater fish, frogs and human [4,6-9]. It is also able to survive freely in freshwater environment [10]. In contrast to human, the body temperatures of freshwater fish and frogs vary with the environmental temperature. The ability to survive in such a wide range of habitats is in line with its ability to survive from 15°C to 42°C, although its growth rate is higher at higher temperatures [8]. In an experiment that examined the differential gene expression of *L. hongkongensis* at 20°C to 37°C using proteomics study, we found that there were 12 differentially expressed protein spots involved in various functions [43]. Seven spots were more highly expressed at 20°C than at 37°C and five more highly expressed at 37°C than at 20°C. Among these were NAGK-37 that was up-regulated at 37°C and NAGK-20 that was up-regulated at 20°C. These two isoenzymes of NAGK catalyze the second step of the arginine biosynthesis pathway.

In addition to the differentially expressed genes detected by 2-dimensional gel electrophoresis, the *L. hongkongensis* genome also contains other genes that could be of importance for adaptation to different temperatures. These include genes related to chaperones and chaperonins, heat shock proteins and cold shock proteins. Overall, the number of CDSs in the *L. hongkongensis* genome encoding putative chaperones and heat shock proteins is lower than that in *C. violaceum*, but higher than those in the *Neisseria* species (Table 10). This phenomenon is similar to that observed in the number of distribution of transcription factors in *L. hongkongensis*, *N. gonorrhoeae*, *N. meningitidis* and *C. violaceum *as described above. On the other hand, the *L. hongkongensis* genome possesses two copies of cspA which encodes cold shock transcription factor and one copy of cspD which encodes cold shock protein homologue (Table 10). There is only one copy of cspA in the genomes of *N. gonorrhoeae*, *N. meningitidis* and *C. violaceum*, whereas cspD is absent from the genomes of *N. gonorrhoeae *and *N. meningitidis*.

**Table 10:** CDSs related to temperature stress in *L. hongkongensis, N. meningitidis, N. gonorrhoeae *and *C. violaceum*

**Product**	**Gene**	***L. hongkongensis *HLHK 9**	***N. meningitidis*** **MC58**	***N. gonorrhoeae*** **FA 1090**	***C. violaceum *ATCC 12472**
Chaperone Hsp40, co-chaperone with DnaK	*dnaJ*	+	+	+	+
Chaperone Hsp70, co-chaperone with DnaJ	*dnaK*	+	+	+	+
Co-chaperone GrpE	*grpE*	+	+	+	+
Chaperone subunit of chaperonin GroEL-GroES	*groEL*	+^a^	+	+	+^a^
Regulator subunit of chaperonin GroEL-GroES	*groES*	+^a^	+	+	+^a^
ATP-dependent protease specificity component and chaperone	*clpA*	+	+	+	+
ClpB chaperone	*clpB*	+	+	+	+
ClpP serine protease	*clpP*	+	+	+	+
Hsc66 chaperone, member of Hsp70 protein family	*hscA*	+	+	+	+
Hsc20 co-chaperone of Hsc66	*hscB*	+	+	+	+
Heat shock protein of Hsp90 family	*htpG*	+	-	-	+
Heat shock protein, integral membrane protein	*htpX*	+	+	+	+^a^
Molecular chaperone Hsp33	*hslO*	+	+	+	+
ATPase component of the HslVU protease	*hslU*	+	-	-	+
Peptidase component of the HslVU protease	*hslV*	+	-	-	+
Heat shock protein Hsp15	*hslR*	+	+	+	+
Cold shock transcription factor	*cspA*	+^a^	+	+	+
Cold shock protein homologue	*cspD*	+	-	-	+

^a^Two copies of the gene are present

### Tolerance to osmotic stress

*L. hongkongensis* can survive in and adapt to a variety of ecological niches, including water and the intestines of freshwater fish, frogs and humans, with different osmotic stress. A total of 25 CDSs in the *L. hongkongensis* genome could be related to control of osmotic pressure (Table 11). Most of these CDSs encode proteins and their regulators for transport of potassium ion, proline and glutamate. Among the 25 CDSs, 11 of them are related to potassium ion transport (nine and two for potassium uptake and efflux respectively); whereas only nine CDSs present in the *C. violaceum *genome and three in the *N. gonorrhoeae *and *N. meningitidis* genomes are related to potassium ion transport (Table 11). In addition to the 11 CDSs related to potassium transport, five other CDSs encode mechanosensitive channel proteins. These channels allow a quick and transient increase in compensatory solute (e.g. proline and glutamate) flux out of bacterial cells in response to large turgor pressure generated by water influx due to osmotic downshock when the bacterial cells are transferred to environments of low osmolarity [69]. Interestingly, a *betT *gene that encodes a transport protein for choline uptake is present in the *L. hongkongensis *genome. However, the *betA *and *betB *genes, that encode enzymes for metabolizing choline to glycine betaine, the osmotically active compound, are absent [70]. Similarly, the *glpR *and *glpK *genes are present. However, the *glpF *gene, another gene in the *glpFK *operon that encodes the glycerol uptake facilitator protein, is absent [71]. Therefore, the contributions of *betT*, *glpR *and *glpK *and their corresponding choline and glycerol transport systems to tolerance of osmotic stress in *L. hongkongensis *are unknown. The expressions of two other CDSs, osmB and osmC, which encode two osmotically inducible lipoproteins, have been found to be affected by change in osmolarity in *E. coli *[72,73]. Both osmB and osmC are membrane proteins of unknown function. In E. coli, it was observed that deletion of osmC will render the bacterium more sensitive to oxidative stress because of its peroxidase activity [74].

**Table 11:** CDSs related to control of osmotic pressure in *L. hongkongensis*, *C. violaceum*, *N. meningitidis *and *N. gonorrhoeae*

Product	Gene	Function	*L. hongkongensis *HLHK 9	*C. violaceum *ATCC 12472	*N. meningitidis *MC58	*N. gonorrhoeae *FA 1090
Sodium/hydrogen exchanger	-	Sodium efflux, hydrogen influx	LHK_00707 LHK_02296	CV2903 CV4147	-	-
Potassium uptake protein	*trkA*	Potassium uptake	LHK_01490	-	NMB1614	NGO1154
Potassium uptake protein	*trkH*	Potassium uptake	LHK_01488	-	NMB0661	NGO0230
Glutathione-regulated potassium-efflux system protein	*kefB*	Potassium efflux, hydrogen influx	LHK_02018	CV3326	NMB0209	-
Glutathione-regulated potassium-efflux system protein	*kef*	Potassium efflux, hydrogen influx	LHK_02848	-	-	NGO1774
Potassium-transporting ATPase, A subunit	*kdpA*	Potassium uptake	LHK_01572	CV1599	-	-
Potassium-transporting ATPase, B subunit	*kdpB*	Potassium uptake	LHK_01573	CV1598	-	-
Potassium-transporting ATPase, C subunit	*kdpC*	Potassium uptake	LHK_01574	CV1597	-	-
Osmosensitive potassium channel signal transduction histidine kinase	*kdpD*	Protein kinase of two-component regulatory system	LHK_01575	CV1596	-	-
Two component transcriptional regulator	*kdpE*	*kdp *operon transcription regulation	LHK_01576	CV1595	-	-
Potassium-transporting ATPase	*kdpF*	Potassium uptake	-	-	-	-
ATP-sensitive inward rectifier potassium channel related transmembrane protein	-	Potassium uptake	-	CV1109	-	-
Low affinity potassium transport system protein	*kup*	Potassium uptake	LHK_01720 LHK_00121	CV2731 CV0573	-	-
Glucose-methanol-choline oxidoreductase	*betA*	Glycine betaine synthesis	-	-	-	-
NAD-dependent betaine aldehyde dehydrogenase	*betB*	Glycine betaine synthesis	-	-	-	-
High-affinity choline transport protein	*betT*	Choline uptake	LHK_01689	CV4302	NMB1277	NGO0529
Large conductance mechanosensitive channel	*mscL*	Compatible solute efflux	LHK_02562	CV1360	-	-
Small conductance mechanosensitive channel	*mscS*	Compatible solute efflux	LHK_01830	CV0295	NMB0042	NGO1771
			LHK_01942	CV2330	NMB0213	NGO2057
			LHK_02394	CV2385		
			LHK_02965	CV2962		
				CV4288		
Osmotically inducible lipoprotein	*osmB*	-	LHK_01892	CV3209	-	-
Osmotically inducible lipoprotein	*osmC*	-	LHK_01612	-	-	-
Sodium glutamate symport carrier protein	*gltS*	Sodium and glutamate uptake	-	CV1105	NMB0085	NGO1890
Proton glutamate symport protein	-	Hydrogen and glutamate uptake	LHK_02672	CV1198 CV3409	-	-
Proline/betaine transproter	*proP*	Proline, glycine betaine and ectoine uptake	LHK_02126	CV1299 CV2901	-	-
ABC-type proline/glycine betaine transport systems, ATPase	*proV*	Proline and glycine betaine uptake	-	CV1197	-	-
Proline-specific permease	*proY*	Proline uptake	-	CV1138	-	-
Osmoprotectant transport system substrate-binding protein	-	Osmoprotectants uptake	-	CV1195 CV4392	-	-
Osmoprotectant transport system permease protein	-	Osmoprotectants uptake	-	CV1194 CV1196 CV4393 CV4395	-	-
Osmoprotectant transport system ATP-binding protein	-	Osmoprotectants uptake	-	CV4394	-	-
Outer membrane porin	*ompC*	Hydrophilic molecules uptake by passive diffusion	-	CV3424	-	-
Outer membrane porin	*ompF*	Hydrophilic molecules uptake by passive diffusion	-	-	-	-
Osmolarity sensor protein	*envZ*	Protein kinase of two-component regulatory system	-	CV0217	-	-
Transcriptional regulator	*ompR*	*ompC *and *ompF *transcription regulation	-	CV0216	-	-
Aquaporin Z	*aqpZ*	Water influx and efflux	-	CV2864	-	-
Glycerol uptake facilitator protein	*glpF*	Glycerol and water uptake	-	CV0252	-	-
Glycerol kinase	*glpK*	Protein kinase of regulatory system	LHK_03100	CV0251	-	-
Glycerol-3-phosphate regulon repressor	*glpR*	Repressor in *glp *operons transcription regulation	LHK_03101	CV0112 CV0136	-	-

### Tolerance to oxidative stress and ultraviolet light stress

Oxidative stress on aerobic bacteria is mainly mediated by partially reduced oxygen species, or reactive oxygen species, most notably superoxide and hydrogen peroxide, that are inevitable by-products of aerobic metabolism. These reactive oxygen species can cause damage to DNA, proteins and membranes. As a result, all aerobic bacteria possess various mechanisms to scavenge superoxide and hydrogen peroxide [75], as well as to protect the cells from damaged by these reactive oxygen species. In most bacteria, inducible responses to superoxide stress and hydrogen peroxide stress are mediated through the transcription factors SoxR(S) and OxyR respectively, which command the induction of a battery of defensive proteins, including superoxide dismutase and catalase respectively [76].

In the *L. hongkongensis *genome, genes for oxidant-resistant dehydratase (*fumC*, *acnA*), superoxide scavenging (*sodB*), hydrogen peroxide scavenging (*ahpC, cpx*), exclusion and export of redox-cycling antibiotics (*acrA, acrB, tolC*), redox balancing (*nfnB*), DNA repair (*xthA, nth, mutM, mutY, mutT*), reduction of disulfide bonds (*trxA, trxB, gpxA, gshA, gshB, grxA, grxC, gor*) [77], limitation of iron availability (*bfr, dps, fur*) and reduction of iron-sulfur clusters (*fpr, yggX*) are present (Table 12). Transcriptions of most of the genes are regulated by SoxR(S) and/or OxyR transcription factors in other bacteria (Table 12) [78]. In addition, some genes may be regulated by other transcription factors, such as RpoS, FNR [79], Fur and Lrp [80,81]. Interestingly, SoxR(S) is not present in the genomes of *N. gonorrhoeae*, *N. meningitidis* and *N. lactamica *and the role of SoxR(S) is presumably taken up by other transcription factors [82]. Notably, SoxR(S) was also not found in the *L. hongkongensis *genome by BLASTp search.

**Table 12:** CDSs related to tolerance of oxidative stress in *L. hongkongensis*, *C. violaceum*, *N. meningitidis *and *N. gonorrhoeae*.

Role	Gene	Protein	Regulated by	*L. hongkongensis *HLHK9	*C. violaceum *ATCC12472	*N. meningitidis *MC58	*N. gonorrhoeae* FA1090
Transcriptional regulator							
*soxR*		SoxR	H_2_O_2_, O_2_	-	CV2793	-	-
*soxS*		SoxS	SoxR	-	-	-	-
*oxyR*		OxyR	H_2_O_2_	LHK_02531	CV3378	NMB0173	NGO1813
*ohrR*		Organic hydroperoxide resistance transcriptional regulator	Organic peroxides	-	CV0210	-	-
*fnr*		Fumarate/nitrate reductase regulator	O_2_	LHK_00352	CV3647	NMB0380	NGO1579
*perR*		PerR	H_2_O_2_	-	-	NMB1266	NGO0542
*lrp*		Leucine-responsive protein	Leucine	LHK_01860	CV1913	NMB0573 NMB1650	NGO1294 NGO1407
Oxidant-resistant dehydratase isozymes							
*fumC*		Fumarase C	SoxRS, RpoS, FNR	LHK_00495	CV1120	NMB1458	NGO1029
*acnA*		Aconitase A	SoxRS, FNR, Fur, RpoS	LHK_02153 LHK_02309	CV1121 CV2054	NMB0433	-
Superoxide scavenging							
*sodA*		Manganese superoxide dismutase	SoxRS, FNR	-	-	-	-
*sodB*		Iron superoxide dismutase		LHK_01716	CV0867 CV2504	NMB0884	NGO0405
*sodC*		Copper-zinc superoxide dismutase	H_2_O_2_, RpoS, FNR	-	-	NMB1398	-
Hydrogen peroxide scavenging							
*ahpC*		Alky hydroperoxide reductase	OxyR, PerR	LHK_00938	CV3739	-	-
*ahpF*		Alky hydroperoxide reductase	OxyR, PerR	-	-	-	-
*cpx*		Cytochrome c peroxidase	FNR	LHK_02666	CV0300	-	NGO1769
catalase/ peroxidase		Hydroperoxidase I	OxyR, RpoS	LHK_01300 LHK_02436	-	-	-
catalase		Hydroperoxidase II	H_2_O_2_, RpoS	LHK_01264	CV3549	NMB0216	NGO1767
Exclusion and export of redox-cycling antibiotics							
*micF*		Antisense RNA to porin OmpF	SoxRS, Lrp, OmpR	-	-	-	-
*acrA-acrB-tolC*		Drug export system	SoxRS	LHK_01426-LHK_01425-LHK_01424	CV0435-CV0434-CV0433	NMB1716-NMB1715-NMB1714	NGO1365-NGO1364-NGO1363
				LHK_02129-LHK_02130-LHK_02131	CV2240-CV2241-CV2242		
				LHK_02929-LHK_02930-LHK_02931			
Redox balancing							
*nfnB*		Nitroreductase	SoxRS	LHK_01953 LHK_03211	CV2244	NMB0804	NGO0388
DNA repair							
*xthA*		Exodeoxyribonuclease III	H_2_O_2_, RpoS	LHK_02447	CV0877	NMB0399	NGO1561
*nth*		Endonuclease III		LHK_01218	CV3293	NMB0533	NGO0139
*nfo*		Endonuclease IV	SoxRS	-	-	-	-
*mutM*		Formamidopyrimidine-DNA glycosylase	FNR	LHK_00316	CV4062	NMB1295	NGO0610
*mutY*		Adenine glycosylase		LHK_02781	CV3703	NMB1396	NGO0710
*mutT*		7,8-dihydro-8-oxoguanine triphosphatase		LHK_02262	CV1787	NMB1064	-
*mutT *homologues		MutT/NUDIX family protein		LHK_00322	CV0032	NMB0453	NGO1506
				LHK_00604	CV1112		
				LHK_01693	CV1586		
				LHK_01823	CV1767		
					CV3401		
					CV3611		
Protein repair							
*msrAB*		Protein-methionine-S-oxide reductase		LHK_01369 (*msrB*)	CV2325 (*msrA*) CV3212 (*msrB*)	NMB0044 (*msrAB*)	NGO2059 (*msrAB*)
Reduction of disulfide bonds							
*trxA*		Thioredoxin 1	ppGpp	LHK_01690	CV1584	NMB1366	NGO0652
-		Thioredoxin		LHK_00591	CV1325	NMB0006	NGO0057
				LHK_01462	CV4257	NMB1845	NGO1923
				LHK_01491	CV4279	NMB1958	NGO2124
				LHK_02476			
				LHK_02092			
*trxC*		Thioredoxin 2	OxyR	-	CV1106	-	-
*trxB*		Thioredoxin reductase		LHK_1482	CV1895 CV2813	NMB1324	NGO0580
-		Peroxiredoxin		LHK_02841	CV3708	NMB0946	NGO0926
*gpxA*		Glutathione peroxidase		LHK_00424	CV1107 CV3555 CV3787	NMB1621	-
*gshA*		Glutamate-cysteine ligase		LHK_03085	CV4276	NMB1037	NGO0608
*gshB*		Glutathione synthase		LHK_03093	CV4275	NMB1559	NGO1217
*grxA*		Glutaredoxin 1	OxyR	LHK_00503	CV3620	NMB0773	NGO0351
*grxB*		Glutaredoxin 2	RpoS, ppGpp	-	-	NMB1734	NGO1381
*grxC*		Glutaredoxin 3		LHK_02837	CV1126	NMB1790	NGO0114
*gor*		Glutathione reductase	OxyR, RpoS, ppGpp	LHK_01492	CV2037	NMB0947	NGO0925
Reduction of iron-sulfur clusters							
*fpr*		NADPH-ferredoxin reductase	SoxRS	LHK_02993	CV0086 CV4045	NMB1044 NMB1450	NGO0687 NGO0734
*fldA*		Flavodoxin	SoxRS	-	-	-	-
*fldB*		Flavodoxin	SoxRS	-	-	-	-
*yggX*		Iron trafficking protein	SoxRS	LHK_00654	CV3356	NMB2021	NGO2083
Organic hydroperoxide resistance							
*ohrA*		Organic hydroperoxide resistance protein	OhrR	-	CV0209	-	-
*ohrB*		Hydrogenperoxide resistance protein	Sigma B	-	CV2493	-	-
Disulfide bond formation in periplasm							
*dsbA*		Disulfide oxidoreductases	Cpx two component system	LHK_02939	CV3998	NMB0278 NMB0294 NMB0407	NGO1548 NGO1717
*dsbB*		Oxidoreductase		LHK_01744	CV3193	NMB1649	NGO1292
*dsbG*		Thiol:disulfide interchange protein	OxyR	-	CV2637	-	-
Increase cellular pools of reduced pyridine nucleotides for glutathione-dependent repair reactions							
*zwf*		Glucose-6-phosphate 1-dehydrogenase	SoxRS	LHK_01919	CV0145	NMB1392	NGO0715
							
Limit iron availability							
*bfr*		Bacterioferritin	Fur	LHK_01239	CV3399 CV3552	NMB1206 NMB1207	NGO0794 NGO0795
*dps*		DNA-binding protein	OxyR, RpoS	LHK_01835 LHK_03179	CV4253	-	-
*fur*		Ferric uptake regulator	PerR, OxyR, SoxRS	LHK_01431	CV1797	NMB0205	NGO1779
Protein binding							
*hslO*		Molecular chaperone Hsp33	H_2_O_2 _& temperature	LHK_02184	CV2000	NMB2000	NGO1189
Others							
*rimK*		Ribosomal protein S6 modification protein	SoxRS	-	-	-	-
*ribA*		Cyclic GMP hydrolase	SoxRS	LHK_02390	CV2005	NMB1254	NGO1134

In addition to oxidative stress, ultraviolet light is another environmental stress that damages the DNA of a bacterium. The genomes of *L. hongkongensis*, *C. violaceum*, *N. gonorrhoeae *and *N. meningitidis *all contain one copy of *phrB *which encodes a photolyase for direct repair of DNA; and one copy each of *uvrA*, *uvrB*, *uvrC *and *uvrD *in the nucleotide excision repair system.

### Starvation related CDSs

*L. hongkongensis *is arguably fastidious: it is asaccharolytic, metabolizing none of the common sugars, requiring malate, adipate or caprate as its carbon source [1,4,43]; in the laboratory, its optimal growth requires brain-heart infusion (BHI) instead of commonplace lysogeny broth (LB) (unpublished data). Thus the pivotal study published in 2007, describing the isolation of *L. hongkongensis *from six of the 10 surveyed drinking water reservoirs in Hong Kong, prompts inquiries into the mechanisms of survival and persistence of this bacterium in nutrient-poor environments [10]. In many natural waters, nutrients are scarce. An average of the reservoirs from which *L. hongkongensis *were isolated demonstrates such: the permanganate value, a surrogate for organic carbon content, had a yearly mean of 1.25 mg O_2_/L; ammoniacal nitrogen, 0.05 mg N/L; and total phosphorus, 0.015 mg P/L [83]. This is in stark contrast with even so-called "minimal medium", in which the malate content measures 2000 mg/L; ammoniacal nitrogen 9.0 mg N/L and total phosphorus 17 mg P/L [84]. Clearly, *L. hongkongensis *has exquisite adaptive abilities which enable its survival in environments such as the drinking water reservoirs.

#### General starvation

With limited nutrients, bacteria do not continue their exponential growth indefinitely. Instead, they move into the stationary phase; cells lose viability and enter the death phase; in prolonged periods of nutrient depletion, a resistant subpopulation survives and the extended stationary phase ensues [85]. To adapt to stress conditions as such, alternative sigma factors enable bacterial RNA polymerase to transcribe an alternative subset of its genes. In the stationary phase, the starvation/stationary phase sigma factor, σ^38^, encoded by *rpoS*, is used to upregulate the expression of a number of genes. Some of these genes may be clustered with *rpoS*: in *L. hongkongensis *a *surE-pcm-nlpD-rpoS *cluster was observed (LHK_00356-00353). This is similar to *C. violaceum *ATCC12472 (CV_3679-3682), and was also observed in other pathogens such as *E. coli*, *Salmonella *Typhimurium [86] and *Yersinia pestis *[87]. In the *L. hongkongensis surE-pcm-nlpD-rpoS *cluster, there is an overlap between the *surE *and *pcm *genes; which was also observed in *C. violaceum*. Despite this overlapping, as shown in *E. coli*, the *pcm *gene can either be co-transcribed with the *surE *gene or transcribed on its own [88].

A CDS coding for the putative gene *surA *precursor is present in the *L. hongkongensis *genome (LHK_03194). This survival protein precursor was also found in *C. violaceum*, *N. meningitidis*, *N. gonorrhoeae *and *E. coli*. SurA, the periplasmic chaperone protein encoded by this gene, is responsible for the proper folding and insertion of a subset of outer membrane proteins in *E. coli *[89]. It is of interest, however, to note that the SurA precursor protein is only expressed at 37°C, but not the environmental temperature of 20°C, when *L. hongkongensis *is cultured in the rich medium of BHI [43]. It is unknown, therefore, whether temperature may have a more generalized effect on the starvation response of *L. hongkongensis*.

#### Carbon starvation

In the *L. hongkongensis *genome, only one CDS coding for the putative carbon starvation gene *cstA2 *was found (LHK_00676). This is similar to *N. gonorrhoeae *and *N. meningitidis*, but different from the *C. violaceum *genome, which contains two CDSs coding for the putative genes *cstA1 *and *cstA2*. The *E. coli *homologue of the *L. hongkongensis cstA2 *gene is *cstA*. CstA is a starvation-induced peptide transporter in *E. coli*, and has been implicated in peptide utilization [90].

CDSs coding for putative genes *sspA *and *sspB *are present in the *L. hongkongensis *genome (LHK02886-02887). Putative homologues of *sspA *and *sspB *are also present in *C. violaceum*, *N. meningitidis*, *N. gonorrhoeae *and *E. coli*. In *E. coli*, *sspA *and *sspB *code for the stringent starvation proteins SspA and SspB. Whilst SspA is essential to expression of SspB, it has also been found to be upregulated in the starvation response to glucose, nitrogen, phosphate and amino acids [91]. SspA and SspB are probably not implicated in the sugar starvation response of *L. hongkongensis*, if any, since the bacterium is asaccharolytic. It is uncertain, nevertheless, whether carbon starvation, i.e. of malate, caprate and adipate, may lead to upregulation of the putative *sspA *and *sspB *genes in *L. hongkongensis*.

#### Phosphorus starvation

It has long been observed that phosphate is often the limiting nutrient of algal and bacterial growth in freshwater environments [92-94]. Bacteria have evolved various mechanisms to enhance the uptake of phosphate, even by cell envelope elongation to increase the surface area to volume ratio [95]; albeit a relationship is yet to be ascribed to the seagull or spiral rod shape of *L. hongkongensis*. From the freshwater reservoir data stated above, phosphate is probably the scarcest nutrient amongst carbon, nitrogen, phosphorus and iron with its concentration of 0.015 mg P/L (or 0.5 μM). On the other hand, however, it is worthwhile to note the more recent finding that phosphate depletion may enhance bacterial resistance to multiple antimicrobials [96,97].

Phosphate homeostasis in bacteria is mainly achieved by the PhoR/PhoB two-component regulatory system (TCRS). In *L. hongkongensis*, the putative genes coding for the PhoR/PhoB are adjacent to each other (LHK_00166-00165), as in *C. violaceum *(CV_0563-0562). The *N. gonorrhoeae *and *N. meningitidis *homologue of the *phoR *and *phoB *genes, however, could not be identified.

The PhoR/PhoB TCRS is closely related to the phosphate-specific transport (Pst) system. In *E. coli*, there is a pstSCAB-phoU operon in which the genes *pstS*, *pstC*, *pstA*, *pstB *and *phoU *are clustered. This is not the case in *L. hongkongensis*, *C. vioalceum*, *N. gonorrhoeae *and *N. meningitidis*. In *L. hongkongensis*, the putative *pstSCAB *locus (LHK00524-00521) is well separated from the CDS coding for the putative *phoU *gene (LHK_00885). In *C. violaceum*, this separation is also seen (*pstSCAB*: CV_0938-0935; *phoU*: CV_1261); the *pstSCAB *locus is also clustered with the putative *pitA *gene, which codes for a low-affinity inorganic phosphate transporter (CV_0934). In contrast to such, the CDS that encodes the putative *pitA *gene in *L. hongkongensis *is separated from the putative *pstSCAB *locus (LHK_02538). It is believed that the PstS, PstC, PstA and PstB proteins, together with PhoU, are responsible for the formation of an ABC transporter in the capture of periplasmic inorganic phosphate. In an abundance of phosphate, the Pst system, together with the histidine kinase PhoR, repress the transcription regulatory protein PhoB. When the extracellular phosphate concentration is below a threshold value, for example 4 mM in *E. coli*, autophosphorylation on a PhoR histidine residue occurs; the phosphorylation is subsequently transferred form phospho-PhoB, which modulates Pho regulon activities [98,99].

## Conclusions

**Figure 2:**
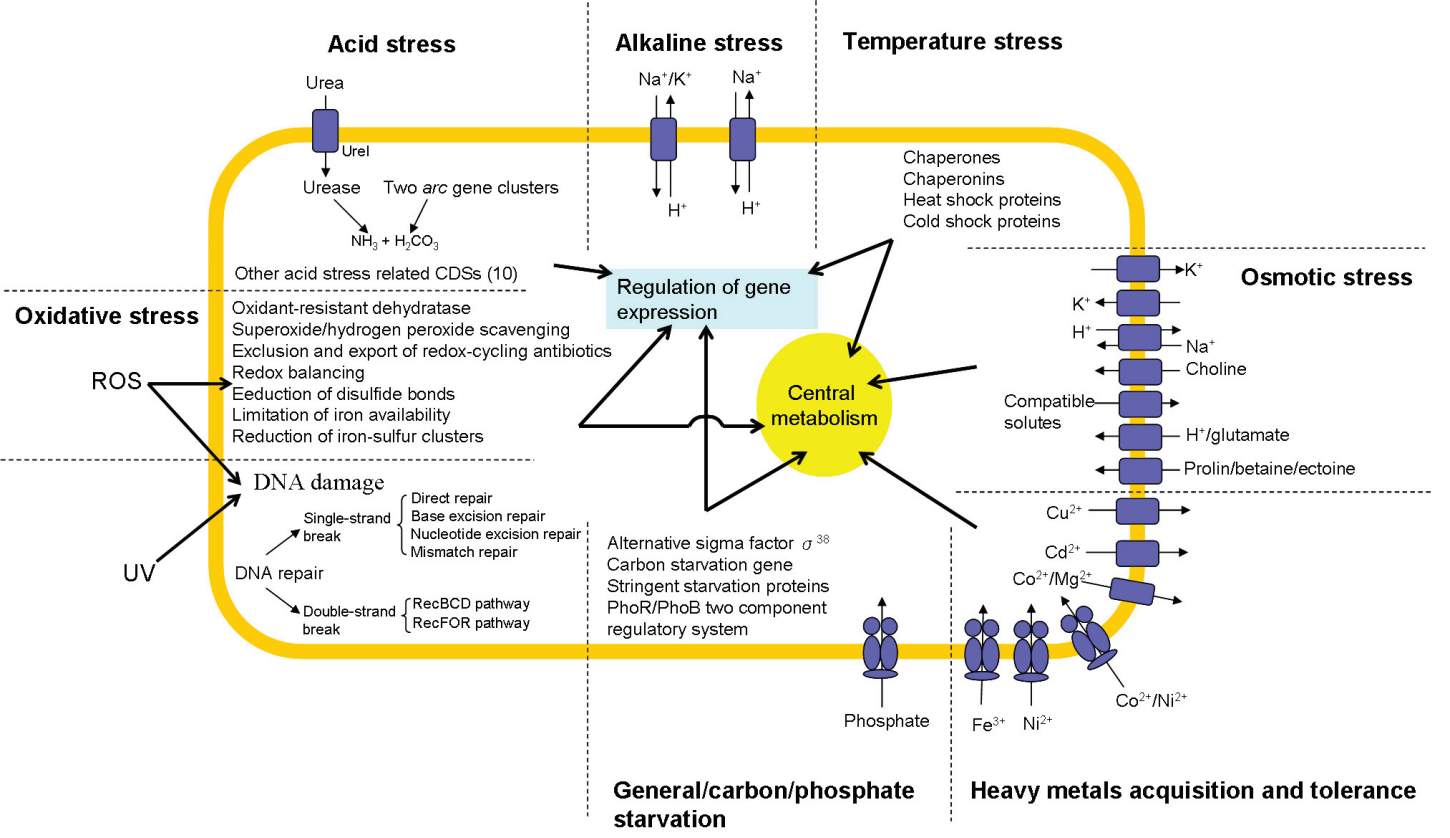
**Metabolic scheme illustrating the mechanisms of response of *Laribacter hongkongensis *to different environment and stress as deduced from its genome sequence**. Different environmental stresses and the corresponding genes in *L. hongkongensis *are shown as indicated (separated by broken lines). Line arrows indicate the flow of pathway. Solid arrows indicate the flow of substances through transporter. Transporters including uniporter, antiporter, symporter, and P-type ATPase are shown as blue rectangular box. ABC transporters are shown as composite figure of 2 circles, 2 ovals and 1 horizontal oval. Integrated view of central metabolism is presented as yellow circle. Integrated view of gene regulation is presented as light blue rectangle. *arc*, arginine deiminase; Cd^2+^, Cadmium(II) ion; Co^2+^, Cobalt(II) ion; Cu^2+^, Copper(II) ion; Fe^3+^, ferrous ion; H^+^, hydrogen ion; H_2_CO_3_, hydrogen carbonate; Mg^2+^, Magnesium(II) ion; Na^+^, Sodium ion; NH_3_, ammonia; Ni^2+^, Nickel(II) ion; K^+^, Potassium ion; ROS, reactive oxygen species; UreI, urea transporter; UV, ultraviolent light.

The *L. hongkongensis *genome possessed a high variety of genes for DNA repair and recombination and regulation of gene expression, as well as adaptation to acid, alkaline, temperature, osmotic, oxidative, UV light and starvation stresses as well as acquisition of and tolerance to heavy metals (Figure 2)

## Methods

All CDSs in the *L. hongkongensis *genome were annotated as described in our previous publication and classified functionally according to the Clusters of Orthologous Groups system [43]. Annotated genes were mapped to pathways according to the Kyoto Encyclopedia of Genes and Genomes database to help identify stress-response pathways. The CDSs were members of COG L (replication, recombination and repair), COG K (transcription), COG F (nucleotide transport and metabolism) and COG O (post-translational modification, protein turnover, chaperones). Additional CDSs were examined by keyword search using the following words and their variants: stress response, regulation, adaptation, temperature, ultraviolet, acid, alkali, pressure, oxidative, homeostasis and resistance. Manual confirmation of the assigned function was performed by sequence similarity search using BLAST against the NCBI nr database, and assisted by conserved domain search (CD-search), identification of signature sequence motifs and sequence analysis using InterProScan. Cellular localization of putative proteins was predicted using PSORTb where appropriate [100]. Phylogenetic relationships were determined using Clustal × version 1.81. *oriC *was predicted by Ori-finder http://tubic.tju.edu.cn/doric/.

## Abbreviations

8oxodG: 7, 8-dihydro-8-oxo-2'-deoxyguanosine; σ24 (RpoE): RNA polymerase sigma-E factor; σ28 (FliA): RNA polymerase sigma factor for flagellar operon; σ32 (RpoH): RNA polymerase sigma-32 factor; σ38 (RpoS): RNA polymerase sigma factor RpoS; σ54 (RpoN): RNA polymerase sigma-54 factor; σ70 (RpoD): RNA polymerase sigma factor; σ-factor(s): Sigma-factor(s); σ-RNA: Sigma-RNA; A: Adenine; ABC: ATP-binding cassette; AcnA: Aconitate hydratase 1; AcrA/B: Acriflavine resistance protein A/B; AhpC: Alkyl hydroperoxide reductase subunit C; AP: Abasic; *argB*: N-acetyl-L-glutamate kinase gene; ATP: Adenosine triphosphate; BER: Base excision repair; BetA: Choline dehydrogenase BetA; BetB: Betaine aldehyde dehydrogenase BetB; BetT: High affinity choline transporter protein BetT; Bfr: Bacterioferritin; BHI: Brain-heart infusion medium; Bp: Base pair; C: Cytosine; CadA: Cadmium efflux ATPase CadA; CDS(s): Coding sequences(s); Cnr: Nickel and cobalt resistance protein Cnr; CopA: Copper-exporting P-type ATPase A; CorA: Magnesium transport protein CorA; CPA: Monovalent cation/proton antiporter; CPA1/2/3: Monovalent cation/proton antiporter-1/2/3; Cpx: Cytochrome peroxidase C; CspA/D: Cold shock protein CspA/D; CstA/B: Carbon starvation-induced protein CstA/B; DNA: Deoxyribonucleic acid;DnaK: Chaperone protein DnaK; DppB/C: Dipeptide transport system permease protein DppB/C; DppD/F: Dipeptide transport ATP-binding protein DppD/F; Dps: Deoxyribonucleic acid protection during starvation protein; DSB(s): Double-strand break(s); DSBR: Double-strand break repair; dsDNA: Double-stranded deoxyribonucleic acid; DUE: DNA-unwinding element; dUMP: Deoxyuridine 5'-monophosphate; dUTP: Deoxyuridine 5'-triphosphate; FbpA: Major ferric iron-binding protein FbpA; FbpB: Ferric transport system permease protein FbpB; FbpC: Ferric ions import ATP-binding protein FbpC; FeoA/B/C: Ferrous iron transport protein A/B/C; Fis: Factor for inversion stimulation; FNR: Fumarate and nitrate reduction regulatory proteins; Fpr: Ferredoxin-NADP reductase; FumC: Fumarase C; Fur: Furric uptake regulation protein Fur; G: Guanine; GlpF: Glycerol uptake facilitator protein; GlpK: Glycerol kinase GlpK; GlpR: Glycerol-3-phosphate regulon repressor protein GlpR; Gor: Glutathione reductase; GO system: 8oxodG system; GpxA: Glutathione peroxidase; GrxA/C: Glutaredoxin-1/3; GshA: Glutamate-cysteine ligase; GshB: Glutathione synthase; HoxN: High-affinity; nickel transport protein HoxN; HtpG: High temperature protein HtpG; IHF(s): Integration host factor(s); Kef: Glutathione-regulated potassium efflux protein Kef; KefB: Glutathione-regulated potassium efflux protein KefB; LB: Lysogeny broth; Lrp: Leucine-responsive regulatory protein; LysR: Transcriptional activator protein LysR; mg N/L: Milligrams of nitrogen per liter; mg O2/L: Milligrams of oxygen per liter; mg P/L: Milligrams of phosphorus per liter; MIT: Metal inorganic transport system; MMR: Mismatch repair; MopA: 60 kDa chaperonin; alternative name for GroEL or Cpn60; mRNA: Messenger ribonucleic acid; MutM: Formamidopyrimidine-DNA glycosylase; MutT: Mutator MutT protein; MutY: Adenine/thymine-specific adenine glycosylase; NAGK: N-acetyl-L-glutamate kinase; Ncc: Nickel-cobalt-cadmium resistance protein Ncc; NER: Nucleotide excision repair; NfnB: Oxygen-insensitive NAD(P)H nitroreductase; NhaA/B/C/D: Sodium/proton antiporter NhaA/B/C/D; NicO/A: High-affinity nickel transport protein NicO/A; NiCoT: Nickel(II)-cobalt(II) uptake transporter; NlpD: Lipoprotein NlpD; NolG: Nodulation protein NolG; Nth: Endonuclease III; O: Oxygen; oriC: Origin of replication; OsmB/C: Osmotically-inducible lipoprotein OsmB/C; OxyR: Hydrogen peroxide-inducible genes regulator OxyR; Pcm: Protein-L-isoaspartate O-methyltransferase; PhoB: Phosphate regulon transcriptional regulatory protein PhoB; PhoR: Phosphate regulon sensor protein PhoR; PhoU: Phosphate transport system protein PhoU; PhrB: Deoxyribodipyrimidine photo-lyase; PitA: Low-affinity inorganic phosphate transporter PitA; Pst: Phosphate-specific transport system; PstS/C/A/B: Phosphate-specific transport system protein S/C/A/B; RIDA: Regulatory inactivation of DnaA; RNA: Ribonucleic acid; RNAP: Deoxyribonucleic acid-directed ribonucleic acid polymerase; RpoS: RNA polymerase sigma factor RpoS; SodB: Superoxide dismutase SodB; SoxR: Redox-sensitive transcriptional activator SoxR; SSAP(s): Single-stranded deoxyribonucleic acid annealing protein(s); ssDNA: Single-stranded deoxyribonucleic acid; SspA/B: Stringent starvation protein SspA/B; SurA/E: Stationary-phase survival protein SurA/E; T: Thymine; TCRS: Two-component regulatory system; TLS: Translesion deoxyribonucleic acid synthesis; TolC: Outer membrane protein TolC; TRCF: Transcription-repair coupling factor; TrxA: Thioredoxin; TrxB: Thioredoxin reductase; UNG: Uracil deoxyribonucleic acid glycosylase; UreA/C/B: Urease subunit gamma/alpha/beta; UreE/F/G/D/I: Urease accessory protein UreE/F/G/D/I; UV: Ultraviolet; UvrA/B/C/D: UvrABC nucleotide excision repair system protein A/B/C/D; XthA Exodeoxyribonuclease III; YggX: Ferrous-trafficking protein; ZntA: Lead, cadmium, zinc and mercury-transporting ATPase ZntA

## Competing interests

The authors declare that they have no competing interests.

## Authors' contributions

PCYW, KYY and SKPL designed and supervised the study. RYYF, TCCH, GKMW, AKLT, JLLT, WC, RMW and SOTC annotated the genome. HT performed bioinformatics analysis. SKPL, RYYF, TCCH, RMW and PCYW drafted the manuscript. All authors corrected the manuscript. All authors read and approved the final manuscript.

## References

[B1] Yuen KY Woo PCY Teng JLL Leung KW Wong MKM Lau SKP *Laribacter hongkongensis *gen. nov., sp. nov., a novel Gram-negative bacterium isolated from a cirrhotic patient with bacteremia and empyema J Clin Microbiol 2001 39 4227 4232 10.1128/JCM.39.12.4227-4232.2001 PMC88529 11724825

[B2] Lau SKP Woo PCY Hui WT Li MWS Teng JLL Que TL Yung RWH Luk WK Lai RWM Yuen KY Use of cefoperazone MacConkey agar for selective isolation of *Laribacter hongkongensis* J Clin Microbiol 2003 41 4839 4841 10.1128/JCM.41.10.4839-4841.2003 PMC254358 14532237

[B3] Woo PCY Kuhnert P Burnens AP Teng JLL Lau SKP Que TL Yau HH Yuen KY Laribacter hongkongensis: a potential cause of infectious diarrhea Diagn Microbiol Infect Dis 2003 47 551 556 10.1016/s0732-8893(03)00161-5 14711474

[B4] Woo PCY Lau SKP Teng JLL Que TL Yung RWH Luk WK Lai RWM Hui WT Wong SSY Yau HH Yuen KY Association of *Laribacter hongkongensis *in community-acquired gastroenteritis with travel and eating fish: a multicentre case-control study Lancet 2004 363 1941 1947 10.1016/S0140-6736(04)16407-6 15194253

[B5] Woo PCY Lau SKP Teng JLL Yuen KY Current status and future directions of *Laribacter hongkongensis*, a novel bacterium associated with gastroenteritis and traveller's diarrhoea Curr Opin Infect Dis 2005 18 413 419 10.1097/01.qco.0000180162.76648.c9 16148528

[B6] Teng JLL Woo PCY Ma SSL Sit THC Ng LT Hui WT Lau SKP Yuen KY Ecoepidemiology of *Laribacter hongkongensis*, a novel bacterium associated with gastroenteritis J Clin Microbiol 2005 43 919 922 10.1128/JCM.43.2.919-922.2005 PMC548085 15695706

[B7] Lau SKP Woo PCY Fan RYY Lee RCM Teng JLL Yuen KY Seasonal and tissue distribution of *Laribacter hongkongensis*, a novel bacterium associated with gastroenteritis, in retail freshwater fish in Hong Kong Int J Food Microbiol 2007 113 62 66 10.1016/j.ijfoodmicro.2006.07.017 16996630

[B8] Lau SKP Lee LCK Fan RYY Teng JLL Tse CWS Woo PCY Yuen KY Isolation of *Laribacterhongkongensis*, a novel bacterium associated with gastroenteritis, from Chinese tiger frog Int J Food Microbiol 2009 129 78 82 10.1016/j.ijfoodmicro.2008.10.021 19033083

[B9] Feng JL Yan H Chowdhury N Neogi SB Yamasaki S Shi L Hu J Chen Q Identification and characterization of integron-associated antibiotic resistant *Laribacter hongkongensis *isolated from aquatic products in China Int J Food Microbiol 2011 144 337 341 10.1016/j.ijfoodmicro.2010.10.014 21075469

[B10] Lau SKP Woo PCY Fan RYY Ma SSL Hui WT Au SY Chan LL Chan JYF Lau ATK Leung KY Pun TCT She HHL Wong CY Wong LLL Yuen KY Isolation of *Laribacter hongkongensis*, a novel bacterium associated with gastroenteritis, from drinking water reservoirs in Hong Kong J Appl Microbiol 2007 103 507 515 10.1111/j.1365-2672.2006.03263.x 17714383

[B11] Parkhill J Wren BW Mungall K Ketley JM Churcher C Basham D Chillingworth T Davies RM Feltwell T Holroyd S Jagels K Karlyshev AV Moule S Pallen MJ Penn CW Quail MA Rajandream MA Rutherford KM van Vliet AH Whitehead S Barrell BG The genome sequence of the food-borne pathogen *Campylobacter jejuni *reveals hypervariable sequences Nature 2000 403 665 668 10.1038/35001088 10688204

[B12] Tettelin H Saunders NJ Heidelberg J Jeffries AC Nelson KE Eisen JA Ketchum KA Hood DW Peden JF Dodson RJ Nelson WC Gwinn ML DeBoy R Peterson JD Hickey EK Haft DH Salzberg SL White O Fleischmann RD Dougherty BA Mason T Ciecko A Parksey DS Blair E Cittone H Clark EB Cotton MD Utterback TR Khouri H Qin H Complete genome sequence of *Neisseria meningitidis *serogroup B strain MC58 Science 2000 287 1809 1815 10.1126/science.287.5459.1809 10710307

[B13] Brazilian National Genome Project Consortium The complete genome sequence of *Chromobacterium violaceum *reveals remarkable and exploitable bacterial adaptability Proc Natl Acad Sci USA 2003 100 11660 11665 10.1073/pnas.1832124100 PMC208814 14500782

[B14] Davidsen T Tønjum T Meningococcal genome dynamics Nat Rev Microbiol 2006 4 11 22 10.1038/nrmicro1324 16357857

[B15] Duarte FT Carvalho FM Bezerra e Silva U Scortecci KC Blaha CA Agnez-Lima LF Batistuzzo de Medeiros SR DNA repair in *Chromobacterium violaceum* Genet Mol Res 2004 3 167 180 15100997

[B16] Michaels ML Cruz C Grollman AP Miller JH Evidence that MutY and MutM combine to prevent mutations by an oxidatively damaged form of guanine in DNA Proc Natl Acad Sci USA 1992 89 7022 7025 10.1073/pnas.89.15.7022 PMC49637 1495996

[B17] Fowler RG White SJ Koyama C Moore SC Dunn RL Schaaper RM Interactions among the *Escherichia coli mutT*, *mutM*, and *mutY *damage prevention pathways DNA Repair (Amst) 2003 2 159 173 10.1016/s1568-7864(02)00193-3 12531387

[B18] Eisen JA Hanawalt PC A phylogenomic study of DNA repair genes, proteins, and processes Mutat Res 1999 435 171 213 10.1016/s0921-8777(99)00050-6 PMC3158673 10606811

[B19] Batty DP Wood RD Damage recognition in nucleotide excision repair of DNA Gene 2000 241 193 204 10.1016/s0378-1119(99)00489-8 10675030

[B20] Seeberg E Reconstitution of an *Escherichia coli *repair endonuclease activity from the separated *uvrA*+ and *uvrB+/uvrC+ *gene products Proc Natl Acad Sci USA 1978 75 2569 2573 10.1073/pnas.75.6.2569 PMC392603 351611

[B21] Black CG Fyfe JA Davies JK Cloning, nucleotide sequence and transcriptional analysis of the *uvrA *gene from *Neisseria gonorrhoeae* Mol Gen Genet 1997 254 479 485 10.1007/pl00008608 9197406

[B22] Marti TM Kunz C Fleck O DNA mismatch repair and mutation avoidance pathways J Cell Physiol 2002 191 28 41 10.1002/jcp.10077 11920679

[B23] Lamers MH Perrakis A Enzlin JH Winterwerp HH de Wind N Sixma TK The crystal structure of DNA mismatch repair protein MutS binding to a G × T mismatch Nature 2000 407 711 717 10.1038/35037523 11048711

[B24] Richardson AR Yu Z Popovic T Stojiljkovic I Mutator clones of *Neisseria meningitidis *in epidemic serogroup A disease Proc Natl Acad Sci USA 2002 99 6103 6107 10.1073/pnas.092568699 PMC122909 11983903

[B25] Mehr IJ Seifert HS Differential roles of homologous recombination pathways in *Neisseria gonorrhoeae *pilin antigenic variation, DNA transformation and DNA repair Mol Microbiol 1998 30 697 710 10.1046/j.1365-2958.1998.01089.x 10094619

[B26] Kline KA Sechman EV Skaar EP Seifert HS Recombination, repair and replication in the pathogenic *Neisseriae*: the 3 R's of molecular genetics of two human-specific bacterial pathogens Mol Microbiol 2003 50 3 13 10.1046/j.1365-2958.2003.03679.x 14507359

[B27] Kline KA Seifert HS Role of the Rep helicase gene in homologous recombination in *Neisseria gonorrhoeae* J Bacteriol 2005 187 2903 2907 10.1128/JB.187.8.2903-2907.2005 PMC1070387 15805536

[B28] Kuzminov A Recombinational repair of DNA damage in *Escherichia coli *and bacteriophage lambda Microbiol Mol Biol Rev 1999 63 751 813 10.1128/mmbr.63.4.751-813.1999 PMC98976 10585965

[B29] Iyer LM Koonin EV Aravind L Classification and evolutionary history of the single-strand annealing proteins, RecT, Redbeta, ERF and RAD52 BMC Genomics 2002 3 8 10.1186/1471-2164-3-8 PMC101383 11914131

[B30] Matsumoto T Morimoto Y Shibata N Kinebuchi T Shimamoto N Tsukihara T Yasuoka N Roles of functional loops and the C-terminal segment of a single-stranded DNA binding protein elucidated by X-Ray structure analysis J Biochem 2000 127 329 335 10.1093/oxfordjournals.jbchem.a022611 10731701

[B31] Yuzhakov A Kelman Z O'Donnell M Trading places on DNA--a three-point switch underlies primer handoff from primase to the replicative DNA polymerase Cell 1999 96 153 163 10.1016/s0092-8674(00)80968-x 9989506

[B32] Courcelle J Khodursky A Peter B Brown PO Hanawalt PC Comparative gene expression profiles following UV exposure in wild-type and SOS-deficient *Escherichia coli* Genetics 2001 158 41 64 10.1093/genetics/158.1.41 PMC1461638 11333217

[B33] Fyfe JA Davies JK Nucleotide sequence and expression in *Escherichia coli *of the *recA *gene of *Neisseria gonorrhoeae* Gene 1990 93 151 156 10.1016/0378-1119(90)90151-g 2121608

[B34] Black CG Fyfe JA Davies JK Absence of an SOS-like system in *Neisseria gonorrhoeae* Gene 1998 208 61 66 10.1016/s0378-1119(97)00653-7 9479048

[B35] Parkhill J Achtman M James KD Bentley SD Churcher C Klee SR Morelli G Basham D Brown D Chillingworth T Davies RM Davis P Devlin K Feltwell T Hamlin N Holroyd S Jagels K Leather S Moule S Mungall K Quail MA Rajandream MA Rutherford KM Simmonds M Skelton J Whitehead S Spratt BG Barrell BG Complete DNA sequence of a serogroup A strain of *Neisseria meningitidis *Z2491 Nature 2000 404 502 506 10.1038/35006655 10761919

[B36] Mott ML Berger JM DNA replication initiation: mechanisms and regulation in bacteria Nat Rev Microbiol 2007 5 343 354 10.1038/nrmicro1640 17435790

[B37] Schmid MB More than just "histone-like" proteins Cell 1990 63 451 453 10.1016/0092-8674(90)90438-k 2121364

[B38] Lorenz M Hillisch A Goodman SD Diekmann S Global structure similarities of intact and nicked DNA complexed with IHF measured in solution by fluorescence resonance energy transfer Nucleic Acids Res 1999 27 4619 4625 10.1093/nar/27.23.4619 PMC148750 10556318

[B39] Wold S Crooke E Skarstad K The *Escherichia coli *Fis protein prevents initiation of DNA replication from *oriC *in vitro Nucleic Acids Res 1996 24 3527 3532 10.1093/nar/24.18.3527 PMC146119 8836178

[B40] Hiasa H Marians KJ Fis cannot support *oriC *DNA replication in vitro J Biol Chem 1994 269 24999 25003 7929185

[B41] Kato J Katayama T Hda, a novel DnaA-related protein, regulates the replication cycle in *Escherichia coli* EMBO J 2001 20 4253 4262 10.1093/emboj/20.15.4253 PMC149159 11483528

[B42] Camara JE Skarstad K Crooke E Controlled initiation of chromosomal replication in *Escherichia coli *requires functional Hda protein J Bacteriol 2003 185 3244 3248 10.1128/JB.185.10.3244-3248.2003 PMC154069 12730188

[B43] Woo PCY Lau SKP Tse H Teng JLL Curreem SOT Tsang AKL Fan RYY Wong GKM Huang Y Loman NJ Snyder LAS Cai JJ Huang JD Mak W Pallen MJ Lok S Yuen KY The complete genome and proteome of *Laribacter hongkongensis *reveal potential mechanisms for adaptations to different temperatures and habitats PLoS Genet 2009 5 e1000416 10.1371/journal.pgen.1000416 PMC2652115 19283063

[B44] Seputiene V Motiejūnas D Suziedelis K Tomenius H Normark S Melefors O Suziedeliene E Molecular characterization of the acid-inducible asr gene of *Escherichia coli *and its role in acid stress response J Bacteriol 2003 185 2475 2484 10.1128/JB.185.8.2475-2484.2003 PMC152617 12670971

[B45] Mates AK Sayed AK Foster JW Products of the *Escherichia coli *acid fitness island attenuate metabolite stress at extremely low pH and mediate a cell density-dependent acid resistance J Bacteriol 2007 189 2759 2768 10.1128/JB.01490-06 PMC1855797 17259322

[B46] Foster JW Bearson B Acid-sensitive mutants of *Salmonella typhimurium *identified through a dinitrophenol lethal screening strategy J Bacteriol 1994 176 2596 2602 10.1128/jb.176.9.2596-2602.1994 PMC205397 8169207

[B47] Chan YC Blaschek HP Comparative analysis of Shigella boydii 18 foodborne outbreak isolate and related enteric bacteria: role of *rpoS *and *adiA *in acid stress response J Food Prot 2005 68 521 527 10.4315/0362-028x-68.3.521 15771176

[B48] Lee IS Lin J Hall HK Bearson B Foster JW The stationary-phase sigma factor sigma S (RpoS) is required for a sustained acid tolerance response in virulent *Salmonella typhimurium* Mol Microbiol 1995 17 155 167 10.1111/j.1365-2958.1995.mmi_17010155.x 7476202

[B49] Yang Y Harris DP Luo F Wu L Parsons AB Palumbo AV Zhou J Characterization of the *Shewanella oneidensis *Fur gene: roles in iron and acid tolerance response BMC Genomics 2008 9 Suppl 1 11 10.1186/1471-2164-9-S1-S11 PMC2386053 18366600

[B50] Padan E Bibi E Ito M Krulwich TA Alkaline pH homeostasis in bacteria: new insights Biochim Biophys Acta 2005 1717 67 88 10.1016/j.bbamem.2005.09.010 PMC3072713 16277975

[B51] Krulwich TA Hicks DB Ito M Cation/proton antiporter complements of bacteria: why so large and diverse? Mol Microbiol 2009 74 257 260 10.1111/j.1365-2958.2009.06842.x PMC2765581 19682259

[B52] Ito M Guffanti AA Zemsky J Ivey DM Krulwich TA Role of the *nhaC*-encoded Na+/H+ antiporter of alkaliphilic *Bacillus firmus *OF4 J Bacteriol 1997 179 3851 3857 10.1128/jb.179.12.3851-3857.1997 PMC179192 9190799

[B53] Nies DH Microbial heavy-metal resistance Appl Microbiol Biotechnol 1999 51 730 750 10.1007/s002530051457 10422221

[B54] Harrison PM Arosio P The ferritins: molecular properties, iron storage function and cellular regulation Biochim Biophys Acta 1996 1275 161 203 10.1016/0005-2728(96)00022-9 8695634

[B55] Anderson DS Adhikari P Nowalk AJ Chen CY Mietzner TA The hFbpABC transporter from *Haemophilus influenzae *functions as a binding-protein-dependent ABC transporter with high specificity and affinity for ferric iron J Bacteriol 2004 186 6220 6229 10.1128/JB.186.18.6220-6229.2004 PMC515168 15342592

[B56] Bauerfeind P Garner RM Mobley LT Allelic exchange mutagenesis of *nixA *in *Helicobacter pylori *results in reduced nickel transport and urease activity Infect Immun 1996 64 2877 2880 10.1128/iai.64.7.2877-2880.1996 PMC174160 8698529

[B57] Morton DJ Seale TW Vanwagoner TM Whitby PW Stull TL The dppBCDF gene cluster of *Haemophilus influenzae*: Role in heme utilization BMC Res Notes 2009 2 166 10.1186/1756-0500-2-166 PMC2738685 19703293

[B58] Liesegang H Lemke K Siddiqui RA Schlegel HG Characterization of the inducible nickel and cobalt resistance determinant *cnr *from pMOL28 of *Alcaligenes eutrophus *CH34 J Bacteriol 1993 175 767 778 10.1128/jb.175.3.767-778.1993 PMC196216 8380802

[B59] Schmidt T Schlegel HG Combined nickel-cobalt-cadmium resistance encoded by the *ncc *locus of *Alcaligenes xylosoxidans *31A J Bacteriol 1994 176 7045 7054 10.1128/jb.176.22.7045-7054.1994 PMC197079 7961470

[B60] Eitinger T Suhr J Moore L Smith JA Secondary transporters for nickel and cobalt ions: theme and variations Biometals 2005 18 399 405 10.1007/s10534-005-3714-x 16158232

[B61] Kobayashi M Shimizu S Cobalt proteins Eur J Biochem 1999 261 1 9 10.1046/j.1432-1327.1999.00186.x 10103026

[B62] Rodionov DA Hebbeln P Gelfand MS Eitinger T Comparative and functional genomic analysis of prokaryotic nickel and cobalt uptake transporters: evidence for a novel group of ATP-binding cassette transporters J Bacteriol 2006 188 317 327 10.1128/JB.188.1.317-327.2006 PMC1317602 16352848

[B63] Nucifora G Chu L Misra TK Silver S Cadmium resistance from *Staphylococcus aureus *plasmid pI258 *cadA *gene results from a cadmium-efflux ATPase Proc Natl Acad Sci USA 1989 86 3544 3548 10.1073/pnas.86.10.3544 PMC287174 2524829

[B64] Zhang Y Zhang H Li X Su Z Zhang C The *cadA *gene in cadmium-resistant bacteria from cadmium-polluted soil in the Zhangshi area of Northeast China Curr Microbiol 2008 56 236 239 10.1007/s00284-007-9064-x 18176824

[B65] Legatzki A Grass G Anton A Rensing C Nies DH Interplay of the Czc system and two P-type ATPases in conferring metal resistance to *Ralstonia metallidurans* J Bacteriol 2003 185 4354 4361 10.1128/JB.185.15.4354-4361.2003 PMC165768 12867443

[B66] Rensing C Sun Y Mitra B Rosen BP Pb(II)-translocating P-type ATPases J Biol Chem 1998 273 32614 32617 10.1074/jbc.273.49.32614 9830000

[B67] Rensing C Mitra B Rosen BP The *zntA *gene of *Escherichia coli *encodes a Zn (II)-translocating P-type ATPase Proc Natl Acad Sci USA 1997 94 14326 14331 10.1073/pnas.94.26.14326 PMC24962 9405611

[B68] Rensing C Fan B Sharma R Mitra B Rosen BP CopA: An *Escherichia coli *Cu(I)-translocating P-type ATPase Proc Natl Acad Sci USA 2000 97 652 656 10.1073/pnas.97.2.652 PMC15385 10639134

[B69] Perozo E Rees DC Structure and mechanism in prokaryotic mechanosensitive channels Curr Opin Struct Biol 2003 13 432 442 10.1016/s0959-440x(03)00106-4 12948773

[B70] Sleator RD Hill C Bacterial osmoadaptation: the role of osmolytes in bacterial stress and virulence FEMS Microbiol Rev 2002 26 49 71 10.1111/j.1574-6976.2002.tb00598.x 12007642

[B71] Weissenborn DL Wittekindt N Larson TJ Structure and regulation of the *glpFK *operon encoding glycerol diffusion facilitator and glycerol kinase of *Escherichia coli *K-12 J Biol Chem 1992 267 6122 6131 1372899

[B72] Jung JU Gutierrez C Villarejo MR Sequence of an osmotically inducible lipoprotein gene J Bacteriol 1989 171 511 520 10.1128/jb.171.1.511-520.1989 PMC209616 2644204

[B73] Gutierrez C Devedjian JC Osmotic induction of gene *osmC *expression in *Escherichia coli *K12 J Mol Biol 1991 220 959 973 10.1016/0022-2836(91)90366-e 1715407

[B74] Conter A Gangneux C Suzanne M Gutierrez C Survival of *Escherichia coli *during long-term starvation: effects of aeration, NaCl, and the *rpoS *and *osmC *gene products Res Microbiol 2001 152 17 26 10.1016/s0923-2508(00)01164-5 11281321

[B75] Zeller T Klug G Thioredoxins in bacteria: functions in oxidative stress response and regulation of thioredoxin genes Naturwissenschaften 2006 93 259 266 10.1007/s00114-006-0106-1 16555095

[B76] Mongkolsuk S Helmann JD Regulation of inducible peroxide stress responses Mol Microbiol 2002 45 9 15 10.1046/j.1365-2958.2002.03015.x 12100544

[B77] Fernandes AP Holmgren A Glutaredoxins: glutathione-dependent redox enzymes with functions far beyond a simple thioredoxin backup system Antioxid Redox Signal 2004 6 63 74 10.1089/152308604771978354 14713336

[B78] Imlay JA Celluar defenses againsts superoxide and hydrogen peroxide Annu Rev Biochem 2008 77 755 776 10.1146/annurev.biochem.77.061606.161055 PMC3057177 18173371

[B79] Kiley PJ Beinert H Oxygen sensing by the global regulator, FNR: the role of the iron-sulfur cluster FEMS Microbiol Rev 1998 22 341 352 10.1111/j.1574-6976.1998.tb00375.x 9990723

[B80] Chen S Rosner MH Calvo JM Leucine-regulated self-association of leucine-responsive regulatory protein (Lrp) from *Escherichia coli* J Mol Biol 2001 312 625 635 10.1006/jmbi.2001.4955 11575919

[B81] Storz G Imlay JA Oxidative stress Curr Opin Microbiol 1999 2 188 194 10.1016/s1369-5274(99)80033-2 10322176

[B82] Seib KL Wu HJ Kidd SP Apicella MA Jennings MP McEwan AG Defenses against oxidative stress in *Neisseria gonorrhoeae*: a system tailored for a challenging environment Microbiol Mol Biol Rev 2006 70 344 361 10.1128/MMBR.00044-05 PMC1489540 16760307

[B83] Personal communication (for data not available online) http://www.wsd.gov.hk/en/water_resources/water_quality/water_quality_monitoring_data/index.html

[B84] Yamamoto K Kato J Yano T Ohtake H Kinetics and modeling of hexavalent chromium reduction in *Enterobacter cloacae* Biotechnol Bioeng 1993 41 129 133 10.1002/bit.260410117 18601254

[B85] Finkel SE Long-term survival during stationary phase: evolution and the GASP phenotype Nat Rev Microbiol 2006 4 113 120 10.1038/nrmicro1340 16415927

[B86] Pappachan A Savithri HS Murthy MR Structural and functional studies on a mesophilic stationary phase survival protein (Sur E) from *Salmonella *Typhimurium FEBS J 2008 275 5855 5864 10.1111/j.1742-4658.2008.06715.x 19021761

[B87] Tidhar A Flashner Y Cohen S Levi Y Zauberman A Gur D Aftalion M Elhanany E Zvi A Shafferman A Mamroud E The NlpD lipoprotein is a novel *Yersinia pestis *virulence factor essential for the development of plague PLoS One 2009 4 e7023 10.1371/journal.pone.0007023 PMC2736372 19759820

[B88] Li C Wu PY Hsieh M Growth-phase-dependent transcriptional regulation of the *pcm *and *surE *genes required for stationary-phase survival of *Escherichia coli* Microbiology 1997 143 3513 3520 10.1099/00221287-143-11-3513 9387229

[B89] Vertommen D Ruiz N Leverrier P Silhavy TJ Collet JF Characterization of the role of the *Escherichia coli *periplasmic chaperone SurA using differential proteomics Proteomics 2009 9 2432 2443 10.1002/pmic.200800794 PMC4004095 19343722

[B90] Schultz JE Matin A Molecular and functional characterization of a carbon starvation gene of *Escherichia coli* J Mol Biol 1991 218 129 140 10.1016/0022-2836(91)90879-b 1848300

[B91] Williams MD Ouyang TX Flickinger MC Starvation-induced expression of SspA and SspB: the effects of a null mutation in *sspA *on *Escherichia coli *protein synthesis and survival during growth and prolonged starvation Mol Microbiol 1994 11 1029 1043 10.1111/j.1365-2958.1994.tb00381.x 8022275

[B92] Correll DL Phosphorus: a rate limiting nutrient in surface waters Poult Sci 1999 78 674 682 10.1093/ps/78.5.674 10228963

[B93] Pradeep Ram AS Sime-Ngando T Functional responses of prokaryotes and viruses to grazer effects and nutrient additions in freshwater microcosms ISME J 2008 2 498 509 10.1038/ismej.2008.15 18273065

[B94] Gikas GD Tsihrintzis VA Akratos CS Haralambidis G Water quality trends in Polyphytos reservoir, Aliakmon River, Greece Environ Monit Assess 2009 149 163 181 10.1007/s10661-008-0191-z 18278562

[B95] Wagner JK Setayeshgar S Sharon LA Reilly JP Brun YV A nutrient uptake role for bacterial cell envelope extensions Proc Natl Acad Sci USA 2006 103 11772 11777 10.1073/pnas.0602047103 PMC1544245 16861302

[B96] Li Y Zhang Y PhoU is a persistence switch involved in persister formation and tolerance to multiple antibiotics and stresses in *Escherichia coli* Antimicrob Agents Chemother 2007 51 2092 2099 10.1128/AAC.00052-07 PMC1891003 17420206

[B97] Rifat D Bishai WR Karakousis PC Phosphate depletion: a novel trigger for *Mycobacterium tuberculosis *persistence J Infect Dis 2009 200 1126 1135 10.1086/605700 19686042

[B98] Wanner BL Hauska G, Thauer R Phosphorus assimilation and its control of gene expression in *Escherichia coli* The Molecular Basis of Bacterial Metabolism 1990 Springer-Verlag, Heidelberg 152 163 Cited in Lamarche et. al.

[B99] Lamarche MG Wanner BL Crépin S Harel J The phosphate regulon and bacterial virulence: a regulatory network connecting phosphate homeostasis and pathogenesis FEMS Microbiol Rev 2008 32 461 473 10.1111/j.1574-6976.2008.00101.x 18248418

[B100] Yu NY Wagner JR Laird MR Melli G Rey S Lo R Dao P Sahinalp SC Ester M Foster LJ Brinkman FS PSORTb 3.0: improved protein subcellular localization prediction with refined localization subcategories and predictive capabilities for all prokaryotes Bioinformatics 2010 26 1608 1615 10.1093/bioinformatics/btq249 PMC2887053 20472543

